# Brain network changes and cognitive function after cardiac arrest

**DOI:** 10.1093/braincomms/fcae174

**Published:** 2024-05-23

**Authors:** Pardis Zarifkar, Mette Kirstine Wagner, Patrick MacDonald Fisher, Dea Siggaard Stenbæk, Selina Kikkenborg Berg, Gitte Moos Knudsen, Michael E Benros, Daniel Kondziella, Christian Hassager

**Affiliations:** Department of Neurology, Copenhagen University Hospital, Rigshospitalet, 2100 Copenhagen, Denmark; Department of Cardiology, Copenhagen University Hospital, Rigshospitalet, 2100 Copenhagen, Denmark; Department of Drug Design and Pharmacology, University of Copenhagen, 2100 Copenhagen, Denmark; Neurobiology Research Unit, Copenhagen University Hospital, Rigshospitalet, 2100 Copenhagen, Denmark; Neurobiology Research Unit, Copenhagen University Hospital, Rigshospitalet, 2100 Copenhagen, Denmark; Department of Psychology, Faculty of Social Sciences, University of Copenhagen, 2100 Copenhagen, Denmark; Department of Cardiology, Copenhagen University Hospital, Rigshospitalet, 2100 Copenhagen, Denmark; Department of Clinical Medicine, University of Copenhagen, 2100 Copenhagen, Denmark; Neurobiology Research Unit, Copenhagen University Hospital, Rigshospitalet, 2100 Copenhagen, Denmark; Department of Clinical Medicine, University of Copenhagen, 2100 Copenhagen, Denmark; Department of Clinical Medicine, University of Copenhagen, 2100 Copenhagen, Denmark; Copenhagen Research Centre for Biological and Precision Psychiatry, Mental Health Centre Copenhagen, Copenhagen University Hospital, 2870 Copenhagen, Denmark; Department of Neurology, Copenhagen University Hospital, Rigshospitalet, 2100 Copenhagen, Denmark; Department of Clinical Medicine, University of Copenhagen, 2100 Copenhagen, Denmark; Department of Cardiology, Copenhagen University Hospital, Rigshospitalet, 2100 Copenhagen, Denmark; Department of Clinical Medicine, University of Copenhagen, 2100 Copenhagen, Denmark

**Keywords:** cardiac arrest, cognitive dysfunction, brain mapping, neural networks, functional neuroimaging

## Abstract

Survival rates after out-of-hospital cardiac arrest have improved over the past two decades. Despite this progress, long-term cognitive impairment remains prevalent even in those with early recovery of consciousness after out-of-hospital cardiac arrest; however, little is known about the determinants and underlying mechanisms. We utilized the REcovery after cardiac arrest surVIVAL cohort of out-of-hospital cardiac arrest survivors who fully regained consciousness to correlate cognition measurements with brain network changes using resting-state functional MRI and the Montreal Cognitive Assessment at hospital discharge and a comprehensive neuropsychological assessment at three-month follow-up. About half of out-of-hospital cardiac arrest survivors displayed cognitive impairments at discharge, and in most, cognitive deficits persisted at three-month follow-up, particularly in the executive and visuospatial functions. Compared to healthy controls, out-of-hospital cardiac arrest survivors exhibited increased connectivity between resting-state networks, particularly involving the frontoparietal network. The increased connectivity between the frontoparietal and visual networks was associated with less favourable cognitive outcomes (β = 14.0, *P* = 0.01), while higher education seemed to confer some cognitive protection (β = −2.06, *P* = 0.03). In sum, the data highlight the importance of subtle cognitive impairment, also in out-of-hospital cardiac arrest survivors who are eligible for home discharge, and the potential of functional MRI to identify alterations in brain networks correlating with cognitive outcomes.

## Teaser

Zarifkar *et al*. found that survivors of out-of-hospital cardiac arrest eligible for home discharge still face significant cognitive impairments, particularly in executive and visuospatial functions. Increased connectivity between the frontoparietal and visual networks correlates with these poorer cognitive outcomes, while higher education levels may offer some protection.

## Graphical Abstract

**Graphical Abstract:**
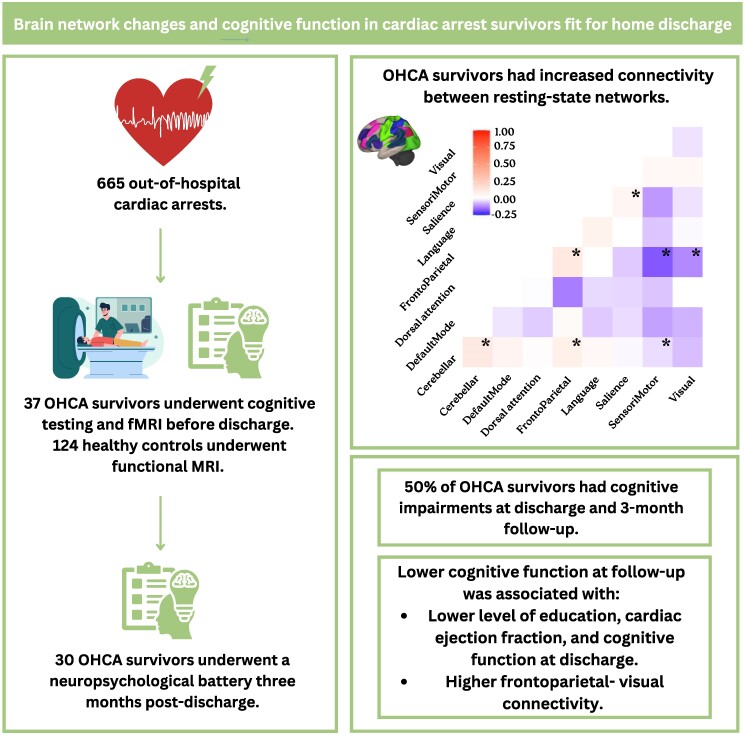

## Introduction

Survival rates for out-of-hospital cardiac arrest (OHCA) have significantly improved in developed nations, showing a 4-fold increase over the past two decades.^1^ Europe and the USA report ∼275 000^2^ and 356 000^3^ OHCA cases annually, with ∼10% of patients surviving until hospital discharge.^4^ However, nearly half of these survivors suffer cognitive decline compared to pre-cardiac arrest, especially regarding memory, executive functions and processing speed, which persists at least up to a year post-discharge.^5-9^ The link between global ischaemia and post-OHCA cognitive effects is well established,^10,11^ yet cognitive dysfunction often goes undetected by standard clinical tools like the Cerebral Performance Category and Modified Rankin Scale.^4,12^ This is particularly observed for survivors without visible structural brain injury on standard neuroimaging, including those eligible for home discharge.^4^

This study, which was part of the REcovery after cardiac arrest surVIVAL (REVIVAL) study,^13,14^ investigated brain network changes using functional MRI (fMRI) correlated with cognitive function in OHCA survivors who were well enough to be discharged home. We hypothesized that OHCA survivors would exhibit distinct cognitive profiles and brain connectivity patterns on fMRI, carrying prognostic implications also in the presence of unremarkable structural brain imaging. Our objectives were 3-fold: to assess cognitive function at discharge and again at three-month follow-up; to compare fMRI profiles between OHCA survivors and healthy controls; and to identify potential demographic, clinical and neuroimaging predictors of cognitive trajectories.

## Materials and methods

### Study design

The REVIVAL study at Copenhagen University Hospital, Rigshospitalet, is a prospective analysis focusing on OHCA survivors ready for home discharge. In a subset of these participants, fMRI was performed.

### Participant enrolment

From January 2018 to February 2022, first-time OHCA survivors of presumed cardiac origin as defined by the Utstein template were enrolled.^15^ The initial study protocol is available for reference.^13^ Eligibility criteria included Danish language proficiency and written informed consent provided 4–9 days post-analgesic-sedation withdrawal. Exclusions were based on MRI contraindications, premorbid neurological, cognitive, or psychiatric conditions, previous cerebrovascular or traumatic brain injuries, a high depression score (>11) on Hospital Anxiety and Depression Scale^16^ and anticipated lack of participation to a three-month follow-up.

Prior to discharge, participants underwent cognitive assessments and neuroimaging. After three months, a comprehensive neuropsychological test battery was administered (see below). Comparative imaging data were included from 124 healthy individuals who underwent identical MRI scans at our institution, accessed via the Cimbi database.^17^

### Clinical and cognitive assessments

Cardiac work-up was done according to standard clinical procedures including echocardiography and percutaneous coronary angiography where indicated. Delirium during admission was assessed using the 4AT scale, a rapid screening tool combining observational and interview-based elements to assess alertness, attention, fluctuations and disorganized thinking.^18^

Functional status at discharge was assessed using three scales: (i) Barthel Index-20^19^: this index quantifies the ability to perform 10 basic activities of daily living, providing a measure of a patients independence and functional status; (ii) Modified Rankin Scale^20^: this scale assesses the degree of disability or dependence in daily activities; and (iii) Cerebral Performance Category Scale^21^: specifically used for cardiac arrest survivors, this scale classifies neurological outcomes ranging from good cerebral performance to death. All patients were provided equal access to physical, psychological and cognitive rehabilitation therapies post-discharge.

Cognitive function was assessed at discharge using the Montreal Cognitive Assessment (MoCA),^22^ adjusted for educational level. A score of 26 or higher was considered indicative of normal cognitive function, while scores below 23 suggested cognitive impairment.^22^ At three-month follow-up, participants’ cognitive functions were reassessed by a health care professional blinded to the participants’ previous data. The neuropsychological test battery assessed episodic memory, executive function, visuospatial construction and verbal fluency as detailed in Supplementary Table 1. Cognitive status was classified as either favourable or unfavourable. We used a conservative criterion, defining clinically significant cognitive impairment as a score ≥ 1.5 SD below the normative mean in two tests within the same cognitive domain, or in at least one test across two or more cognitive domains.^23-27^

### Structural and functional MRI imaging and processing

MRI scans were performed using a Siemens MAGNETOM 3T Prisma scanner with a 64-channel head and neck coil. We acquired a high-resolution, whole-brain T_1_-weighted sequence (MP-RAGE) with the following parameters: inversion time of 900 ms, repetition time of 1900 ms, echo time of 2.58 ms, flip angle of 9°, in-plane matrix of 256 × 256 mm, in-plane resolution of 0.9 × 0.9 mm and 224 slices (0.9 mm slice thickness). Resting-state fMRI (rs-fMRI) scans were acquired using a T_2_*-weighted gradient echo-planar imaging (EPI) sequence with a repetition time of 2000 ms, echo time of 30 ms, flip angle of 90°, in-plane matrix of 64 × 64 mm, in-plane resolution of 3.6 × 3.6 mm, slice thickness of 3 mm with a 0.75 mm gap, 32 slices acquired interleaved, bottom-up. Individuals were scanned for 10 min, i.e. 300 whole-brain volumes were acquired. An accompanying field map was generated to correct spatial distortions in the EPI images. During the rs-fMRI scan, participants were instructed to keep their eyes closed and let their minds wander without falling asleep.

### Preprocessing

Data were preprocessed using SPM12 (https://www.fil.ion.ucl.ac.uk/spm/software/spm12) and spmup toolbox (https://github.com/CPernet/spmup) in MATLAB R2021 (https://www.mathworks.com). Steps included slice-timing correction, spatial realignment and unwarping, co-registration with T_1_-weighted structural images, tissue-type segmentation (based on T_1_-weighted structural images), spatial normalization into Montreal Neurological Institute space (final voxel size 3 × 3 × 3 mm) and smoothing with a 9 mm full-width at half-maximum kernel. Further denoising was performed with CONN v19(RRID:SCR_009550).^28,29^ The time series underwent bandpass filtering (0.008–0.09 Hz), and noise sources were regressed from the time series including anatomical component correction (aCompCor),^30^ motion parameters and outlier volumes, which were identified via Artifact Detection Tools (https://web.mit.edu/swg/software.htm).

### Data analysis

Functional Connectivity analysis was conducted using mean denoised time series extracted from regions of interest (ROIs) defined a priori by the ‘networks’ atlas in CONN, which categorizes 32 regions of the brain into eight resting-state networks (default mode, dorsal attention, frontoparietal, language, salience, sensorimotor, visual and cerebellar networks; Supplementary Fig. 1). Connectivity between ROI pairs was assessed using Fisher’s *r*-to-*z* transformation of Pearson’s rho, i.e. *z* = artanh(*r*), where *r* represents the Pearson’s rho correlation coefficient and ‘artanh’ represents the inverse hyperbolic tangent function. For each scan session, a region-to-region connectivity matrix was compiled. Within-network connectivity was calculated as the mean connectivity across ROI pairs within the same network (i.e. eight within-network measures). Between-network connectivity referred to the mean connectivity across ROI pairs spanning different networks (i.e. 28 between-network pairs). Global functional connectivity was determined as the mean connectivity across all ROI pairs, irrespective of network affiliation.

### Statistical analyses

Differences in demographic and clinical characteristics between OHCA survivors and healthy controls, and between OHCA survivors at follow-up versus those lost to follow-up, were assessed using *t*-tests or Welch’s *t*-tests (*t*), Mann–Whitney U-tests (W), or Chi-squared tests (χ^2^) as appropriate. Differences in functional connectivity were analysed using analysis of covariance (ANCOVA), adjusted for age, sex and education level (0—primary, 1—secondary, 2—tertiary). In sensitivity analyses, demographic matching between groups was refined using propensity score weighting, implemented through the Weightit package in R. This informed a weighted ANCOVA for functional connectivity comparisons. *Post hoc* Bonferroni corrections were applied to the group-level comparisons to adjust for multiple comparison adjustments when analysing differences in functional connectivity between OHCA survivors and healthy controls. Within and between-network connectivity patterns were visualized using heatmaps. To identify predictive factors for cognitive function three months post-discharge, we employed Least Absolute Shrinkage and Selection Operator (LASSO) regression, selecting from a comprehensive set of demographic, clinical and neuroimaging variables. These included AED defibrillations, time to return of spontaneous circulation (ROSC; minutes), cardiac ejection fraction assessed one-week post-arrest (%), targeted temperature management, sedation level (0—none, 1—propofol or remifentanil, 2—propofol or remifentanil and benzodiazepines), coma duration (hours), hospitalization length (days), delirium incidence and MoCA score at discharge, along with global, within- and between-network connectivities. The strength of regularization in LASSO was determined by the optimal lambda parameter, identified via cross-validation within the glmnet framework in R. In a sensitivity analysis, we performed further LASSO regression analyses with lambda values 0.01 units above and below from the optimal value, to evaluate the stability of the selected variables. Post-LASSO, regular logistic regression was conducted between binary cognitive outcomes (favourable/unfavourable) and the selected variables. The logistic regression model was adjusted for demographic factors if not selected by LASSO in sensitivity analyses. Missing data were reported and excluded. All analyses were conducted using R statistical software v. 4.3.2 (R Core Team, Vienna, Austria, 2022).

### Ethical approval

The study adhered to the Declaration of Helsinki and received approval from the regional Danish Research Ethics Committee (H-18046155).

## Results

### Demographic and clinical characteristics

**Figure 1:**
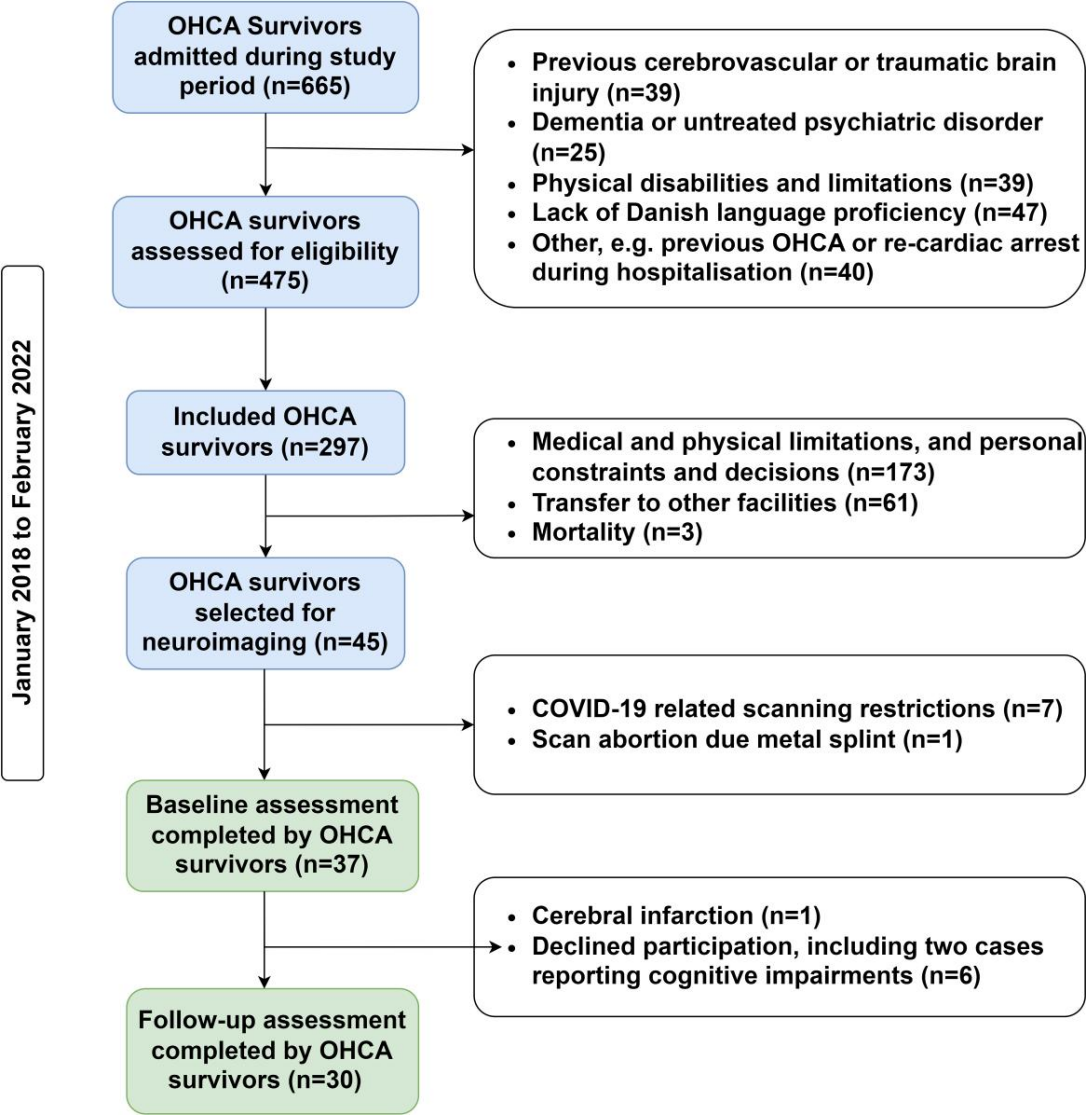
**REVIVAL study participant inclusion flowchart.** This flowchart illustrates the participant selection process for the REVIVAL study, conducted from January 2018 to February 2022. Starting with 665 OHCA survivors, 37 were ultimately included in this sub-study, 30 of whom participated in the three-month follow-up. Details of the exclusion criteria are provided within the chart. COVID-19, coronavirus disease 2019; OHCA, out-of-hospital cardiac arrest.

Between January 2018 and February 2022, we identified 45 eligible OHCA survivors for the REVIVAL fMRI sub-study. Of these, data were not acquired in seven patients due to COVID-19 restrictions and one due to an incidental finding of metal splints, resulting in a final cohort of 37 participants. The participant selection process, including specific exclusion reasons, is detailed in the flowchart (Fig. 1).

Demographic and clinical characteristics are detailed in Table 1. The median age of the final cohort was 53 years (IQR: 20). The majority (*n* = 31, 84%) were male, and eight (22%) had completed tertiary education. At the time of OHCA, 36 (97%) exhibited a shockable rhythm, with initial rhythms of ventricular fibrillation in 33 (81%), pulseless electrical activity in 4 (11%) and unknown in 3 (8%). ROSC was achieved within a mean of 17 min from the emergency call.

**Table 1:** Demographic and clinical characteristics of OHCA survivors at discharge

Demographic		Clinical	
Age	51 ± 14; 53 (IQR: 20)	Cardiovascular and neurological co-morbidities	Hypertension, COPD or CKD: 15 (41%) IHD or AMI: 6 (16%) Migraine: 1 (3%)
Sex (M)	31 (84%)	Past cardiovascular interventions	Percutaneous Coronary Intervention: 3 (8%) Coronary Artery Bypass Graft: 1 (3%)
Education	Primary: 20 (54%) Secondary: 7 (19%) Tertiary: 8 (22%) Unknown: 2 (5%)	Ejection fraction (%)	52 ± 10; 55 (IQR: 10)
Employment status	Full-time: 25 (68%) Maternity, medical leave or part-time: 3 (8%) Retired: 7 (19%) Unknown: 1 (3%)	Awake at arrival	12 (32%)
Functional status at discharge		Shockable rhythm	36 (97%)
Barthel Index	100	OHCA to ROSC (minutes)	17 ± 15; 10 (IQR: 8)
Modified Rankin Scale	Score 1: 35 (95%)	Coma (hours)	26 (IQR: 45)
Cerebral Performance	Score 1: 36 (97%)	ICU (hours) Hospital (days)	53 (IQR: 84) 12 (IQR: 6)
MoCA	25 ± 3; 26 (IQR: 4)	Delirium in the ICU	5 (14%)

AMI, acute myocardial infarction; IHD, ischaemic heart disease; CKD, chronic kidney disease; COPD, chronic obstructive pulmonary disorder; CPR, cardiopulmonary resuscitation; M, male; ICD, implantable cardioverter defibrillator; MoCA, Montreal Cognitive Assessment; OHCA, out-of-hospital cardiac arrest; ROSC, return of spontaneous circulation.

Bystander CPR was administered in 33 (89%) of cases. During ICU stay, 25 (67%) received sedation with propofol, remifentanil and/or benzodiazepines, 22 (59%) underwent targeted temperature management, and 5 (14%) experienced delirium. An implantable cardioverter defibrillator (ICD) was implanted in 35 (96%) of patients. Upon discharge, 36 (97%) of the patients were evaluated as having intact or only mildly impaired neurological function, as indicated by scoring 1 on the Cerebral Performance Scale.

At three-month follow-up, 30 of the 37 OHCA patients were reassessed; median age 53 years (IQR: 19), 26 (87%) males, 8 (27%) with tertiary education. The average time to follow-up was 92 ± 14 days (mean ± standard deviation). The control neuroimaging group comprised 124 healthy individuals, with a median age of 27 (IQR: 8), 52 (42%) were male and 46 (37%) had tertiary education. The OHCA group was significantly older (mean age 51 versus 30, *t* = −8.57, df = 43.769, *P* < 0.001) and showed differences in sex (χ^2^ = 18.3, df = 1, *P* < 0.001) and education distributions (χ^2^ = 40.0, df = 2, *P* < 0.001) compared to controls. No significant differences were observed between patients lost to follow-up and those attending follow-up in terms of age (*t* = −0.31, *P* = 0.77), sex (χ^2^ = 0.17, *P* = 0.68), education (W = 121, *P* = 0.14), baseline MoCA scores (*t* = −0.10, *P* = 0.92), or clinical characteristics (*P* > 0.05).

### Cognitive outcomes and structural neuroimaging in OHCA survivors

At discharge, the mean MoCA score for the OHCA group was 25 ± 3, with scores ranging from 18–29. Nine (24%) scored between 24 and 26 points, indicating possible cognitive impairment, and eight (22%) scored below 23, indicating definite cognitive impairment. No significant associations were found between MoCA scores and age (*r* = 0.10, *P* = 0.56), sex (*t* = −0.77, *P* = 0.46), or education (F = 1.21, *P* = 0.31), suggesting that demographic factors did not have a major influence on cognitive performance as measured by MoCA in our sample.

At three-month follow-up, 15 (50%) OHCA survivors met criteria for unfavourable cognitive outcomes, with impairment in one or more cognitive domains. Specific impairment was observed in executive function (*n* = 19, 32%), visuospatial abilities (*n* = 7, 23%), verbal fluency (*n* = 6, 20%) and episodic memory (*n* = 5, 17%). There were no significant differences in age (*t* = −0.20, *P* = 0.84), sex (χ^2^ = 0.06, *P* = 0.80), or education (W = 151, *P* = 0.07) between cognitively favourable and unfavourable patient groups.

### Resting-state fMRI findings

#### Global connectivity patterns

**Figure 2:**
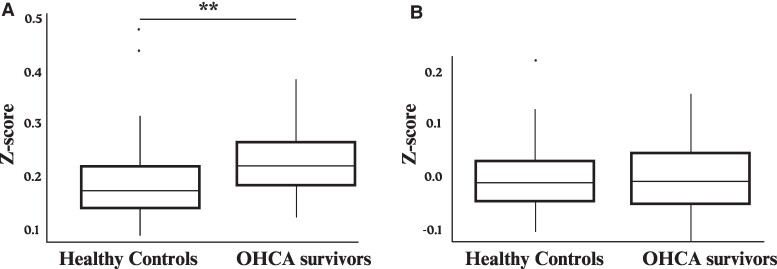
**Global connectivity after out-of-hospital cardiac arrest.** (**A**) Boxplot of the mean Fisher’s *z*-transformed correlation coefficients across all region-of-interest pairs, comparing resting-state global connectivity between out-of-hospital cardiac arrest patients (*n* = 37) and healthy controls (*n* = 124). Welch’s *t*-test indicated higher global connectivity in OHCA survivors compared to healthy controls (W = −2.98, *P* = 0.004). (**B**) Boxplot of the residuals for global connectivity adjusted for age, sex and education demonstrates diminished differences between the groups. Analysis of covariance accounting for demographics showed no significant difference in global connectivity (β = 0.01, *P* = 0.53). The whiskers represent the full range of the data (except for outliers that are indicated by ‘.’), while the boxes show the interquartile range. The horizontal line within each box indicates the median value. **P ≤ 0.01.

Initial analyses indicated higher global connectivity in OHCA survivors compared to healthy controls (Fig. 2A; W = −2.98, *P* = 0.004). After adjusting for demographics (age, sex and education), this difference was not significant (Fig. 2B; β = 0.01, *P* = 0.53). In the adjusted model, sex emerged as a significant factor, with males exhibiting lower global connectivity than females (β = 0.03, *P* = 0.01). Age and education were not significantly associated with global connectivity (age: β = 0.001, *P* = 0.13; education: β = −0.0004, *P* = 0.10). The use of propensity score weighting for demographic variables similarly demonstrated no difference in global connectivity between OHCA survivors and healthy controls (β = 0.004, *P* = 0.82).

### Within-network and between-network connectivities

**Figure 3:**
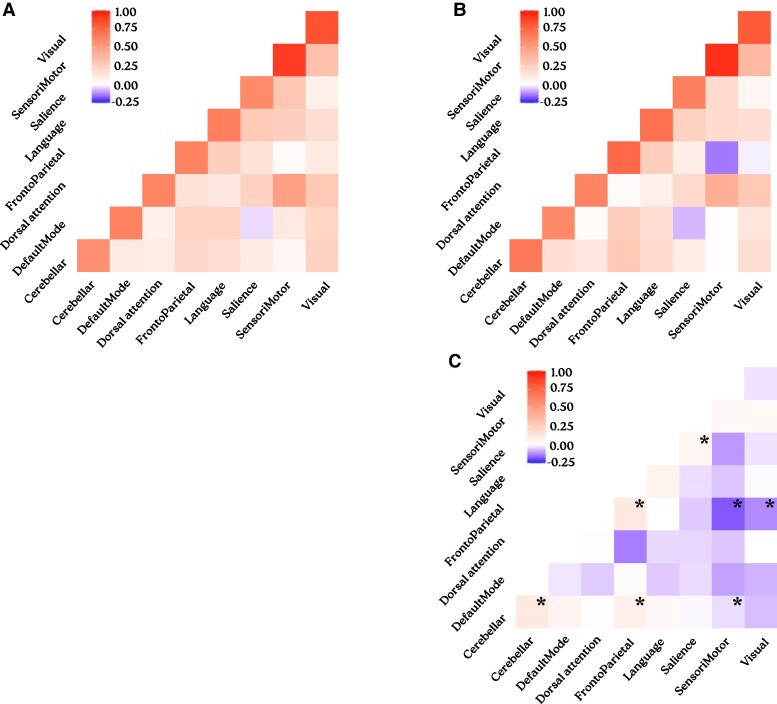
**Resting connectivity in OHCA survivors compared to healthy controls.** Heatmap illustrating Fisher’s *z*-transformed correlation coefficients within and between resting-state networks in OHCA survivors (*n* = 37, **A**) and healthy controls (*n* = 124, **B**). A comparative heatmap shows the differences in network connectivity between OHCA survivors and healthy controls (**C**). The use of propensity score weighting for demographic variables demonstrated significant decreases in within-network connectivity and increases in between-network connectivity, especially involving the frontoparietal network. This pattern suggests a potential compensatory response to maintain cognitive function in the OHCA survivor group. Asterisks (*) denotes significantly altered connectivities after adjustment for multiple testing (*P* < 0.015).

Between-network connectivity was higher in OHCA survivors than controls, especially involving the frontoparietal network. After controlling for demographic variables and adjusting for multiple comparisons, significant increases persisted between the frontoparietal and visual networks (β = 0.14, *P* = 0.01), as well as the frontoparietal and sensorimotor networks (β = 0.17, *P* = 0.01). Decreases in within-network connectivity did not reach statistical significance (*P* > 0.05; Supplementary Table 2). The use of propensity score weighting for demographic variables confirmed increased connectivity between resting-state networks in OHCA survivors compared to healthy controls, particularly involving the frontoparietal and cerebellar networks. Significant decreases in within-network connectivity were also observed for the frontoparietal, dorsal attention, cerebellar and salience networks (Supplementary Table 3; Fig. 3).

### Demographic, clinical and neuroimaging predictors of cognitive outcomes in OHCA survivors

Lasso regression was employed to select relevant variables from a broad set of demographic, clinical and neuroimaging factors potentially influencing cognitive outcomes at follow-up. Significant variables identified were education, cardiac ejection fraction, MoCA score at discharge, along with connectivity between the frontoparietal and visual networks (henceforth referred to as frontoparietal-visual connectivity), and between the sensorimotor and language networks. Sensitivity analyses with decrements in the lambda value included more variables, aligning with LASSO regression’s characteristics where lower lambda values carry lower penalization i.e. a reduction in the magnitude by which the regression coefficients are shrunk towards zero, resulting in higher retention of variables. Conversely, increments from the optimal lambda value consistently selected the same predictors, indicating that these variables significantly contribute to the model’s predictive ability (Supplementary Table 4).

Subsequent logistic regression analysis confirmed the associations of education and frontoparietal-visual connectivity with favourable or unfavourable cognitive outcomes. Specifically, higher education was associated with a reduced risk of unfavourable cognitive outcomes (β = −2.06, *P* = 0.03), whereas increased frontoparietal-visual connectivity was associated with a greater likelihood of unfavourable cognitive outcomes (β = 14.0, *P* = 0.01). Incorporating age and sex as covariates, the influence of education (β = −2.02, *P* = 0.046) and frontoparietal-visual connectivity (β = 13.8, *P* < 0.01) on cognitive outcomes remained significant. The inclusion of these covariates improved the model’s fit, evidenced by a lower an Akaike Information Criterion (AIC) from 33.3 to 30.5.

## Discussion

### Cognitive function over time in OHCA survivors

Our study revealed significant cognitive impairment in OHCA survivors at discharge with 46% scoring below the normal threshold on the MoCA. At three-month follow-up, this impairment persisted, with 50% showing deficits in at least one cognitive domain. The most affected areas were executive and visuospatial functions, but deficits were also found in verbal fluency and episodic memory, highlighting the broad spectrum of cognitive challenges after OHCA. Notably, we found no significant correlation between immediate post-OHCA cognitive performance and demographic variables.

Studies on OHCA report a wide range of cognitive impairment, from 6% to 100% prevalence.^5-9,31-33^ Such variability reflects methodological differences, diverse study populations, small sample sizes and the retrospective nature of most studies.^6,7^ Our findings align with those of similar prospective studies, where approximately half of OHCA survivors are found to be cognitively impaired.^6,7,31-33^ For instance, one study identified neuropsychological deficits in 24 out of 57 (42%) OHCA survivors, mainly in attention and motor skills;^33^ however, the study’s cognitive assessment was limited to the Trail Making Test, without separate measures for each cognitive domain. By contrast, a larger study with 184 OHCA survivors showed that 53 (29%) experienced impairment in at least two cognitive domains, particularly in executive function, memory and processing speed, seven months post-event.^5^ This echoes earlier findings, such as one study reporting cognitive impairments in 50% of 38 OHCA survivors after six months,^31^ and another study observing such impairments in 60% of 57 survivors after three months, with deficits in memory and planning.^33^ Together, these studies underscore the persistence of cognitive challenges in OHCA survivors, despite advances in clinical management and assessment techniques, and highlight the need for cognitive rehabilitation in post-OHCA care.

### Functional reorganization after OHCA

Our study presents fMRI patterns in OHCA survivors well enough for home discharge, a cardiac population not previously explored with functional neuroimaging. Analyses revealed distinctive brain connectivity patterns in OHCA survivors at discharge compared to healthy controls. Although global network connectivity remained intact, we observed a general reduction in within-network connectivity and an increase in between-network connectivity, especially pertaining to the frontoparietal network. The frontoparietal network is important for cognitive control and the coordination of behaviour, enabling rapid, accurate and flexible responses to goal-driven tasks,^34^ and decreased connectivity within the frontoparietal network but increased connectivity between the frontoparietal network and other networks may indicate an inadequate compensatory response to altered brain networks, as observed in other cases of cerebral injury, such as traumatic brain injury or early neurodegenerative disease stages.^35-37^

Demographic differences in age, sex and education carry potential neurobiological implications for fMRI outcomes.^38-40^ In our study, we observed an association between sex and network connectivity, with females exhibiting higher global connectivity than males. This finding is consistent with existing literature that documents both structural^41^ and functional differences in brain connectivity between sexes.^42-48^ Reports of functional differences, however, show greater diversity in their outcomes. For example, a study involving 336 females and 225 males reported higher local functional connectivity density in females.^42^ Another study with 1685 participants from three cohorts, using resting-state connectivity for sex classification, identified distinct functional organization patterns in specific brain regions, but did not investigate global connectivity.^43^ Finally, a study of 2878 participants revealed greater connectivity within the frontoparietal network, dorsal attention network and sensorimotor networks in males,^49^ while another study of 5216 participants reported greater connectivity within the default mode, visual and sensorimotor networks in females.^41^

Changes in functional connectivity are also seen with age. Specifically, a general decrease in global and within-network, especially involving the default mode, ventral attention and sensorimotor networks,^39,49^ and patterns of both increases and decreases in between-network connectivity, especially involving the default mode network.^38-40^ The decrease in connectivity is most marked in individuals aged 65–79 years, followed by an increase after 80 years.^38-40^ This pattern suggests potential compensatory mechanisms, especially in between-network connectivity among older, more educated adults.^38-40,50^ Given these findings, the older, less educated and predominantly male group of OHCA survivors would be expected to have lower global network connectivity, with a pattern of functional integration and segregation centred on the default mode network. Contrary to these expectations, OHCA survivors had a distinct pattern of functional reorganization centred on the frontoparietal network, with unchanged global connectivity. This deviation from brain network connectivity found in normal aging suggests that the increased network connectivity observed in our patients is a result of neuronal reorganization following OHCA, rather than being driven by demographic factors.

### Demographic, clinical and neuroimaging predictors of cognitive function post-OHCA

Education, cardiac ejection fraction, MoCA scores at discharge and frontoparietal-visual connectivity emerged as potential predictors of cognitive outcomes in OHCA survivors. Of these, education level and frontoparietal-visual connectivity were significantly associated with cognitive function at three-month follow-up. The correlation between higher education and more favourable cognitive outcomes is consistent with the cognitive reserve theory that posits that pre-existing cognitive abilities can mitigate the impact of brain injury,^51,52^ and connectivity patterns associated with high cognitive reserve have been linked to better cognitive performance.^50^ By contrast, increased frontoparietal-visual connectivity, possibly indicating an inadequate compensatory response,^35-37,50^ was associated with poorer outcomes, suggesting that fMRI connectivity may have the potential to serve as a biomarker of cognitive function after OHCA.

While cardiac ejection fraction was related to cognitive outcomes, it was not a significant predictor in our study. This observation is consistent with previous research that associates very low ejection fractions with cognitive deficits.^53^ In our cohort, ejection fraction was only moderately decreased, with only 4 (11%) performing under 40%, which may not have been low enough to impact cognitive function. Finally, the MoCA scores at discharge were only loosely correlated with later cognitive outcomes, potentially due to their limited sensitivity in detecting subtle, domain-specific deficits.^54,55^ This finding emphasizes the importance of routine cognitive monitoring post-OHCA using comprehensive neuropsychological test batteries. Hence, OHCA survivors and their families and caregivers should be vigilant about potential delayed cognitive challenges, even if initial screening assessments are normal.

### Strengths and limitations

Our study was novel in its focus on neural connectivity and cognitive outcomes in post-OHCA survivors without overt structural brain injury who were ready for home discharge. However, several limitations must be acknowledged. First, due to logistical reasons, neuroimaging and MoCA were not repeated at three-month follow-up. This precluded a direct comparison of brain network changes and cognitive function over time, a gap that future studies should address. Additionally, our study evaluated only resting-state functional connectivity. Cognition-related task-based fMRI may possibly yield more sensitive results in identifying prognostic brain imaging biomarkers, although this remains to be shown. Second, a more comprehensive assessment at discharge could have revealed domain-specific cognitive impairment in the acute stage. Additionally, a proper psychiatric assessment may have identified mood disorders known to be associated with cognitive deficits.^56^ Third, our sample comprised 37 OHCA survivors enrolled consecutively over nearly five years. Although our sample is both demographically and clinically representative of a general OHCA population and larger than many similar cohorts,^3,4,6,57-59^ it is unlikely to fully represent the entire spectrum of post-OCHA survivors without overt brain injury, for example, the rate of ICD placement was high (96%), potentially introducing a selection bias, where those without an indication for ICD indication after revascularization of obstructive coronary artery disease were underrepresented. Fourth, the pre-OHCA cognitive function of individuals could not be considered due to the inherent unpredictability of the cardiac event. Finally, the control group of 124 healthy participants was not demographically matched to the OHCA cohort. Although we adjusted for age, sex and educational background, this approach has its limitations as already discussed. The cognitive reserve theory posits that while certain predictors indicate potential cognitive decline, actual outcomes are influenced by mitigating factors like education, intelligence and neural plasticity.^60^ Our study considered some of these elements, but future investigations should include larger groups and additional influencing factors. In sum, although our study does not fully capture the disparities in individual patient performances, we believe it nevertheless adds insights into cognitive trajectories post-OHCA by integrating functional imaging, demographic and clinical perspectives.

## Conclusions

OHCA survivors with early recovery of consciousness and no visible structural brain injury can still exhibit substantial cognitive impairment that correlates with alterations in brain network connectivity, specifically increased between-network resting-state connectivity. In almost half of OHCA survivors, cognitive impairment persisted from hospital discharge to three-month follow-up, particularly affecting executive and visuospatial functions. Higher education seemed to confer some cognitive protection, whereas increased connectivity between the frontoparietal and visual networks was associated with less favourable cognitive outcomes. These observations support the cognitive reserve theory and identify a potential fMRI biomarker for predicting post-OHCA cognitive trajectories.

## Supplementary Material

fcae174_Supplementary_Data

## Data Availability

Anonymized data are available from the corresponding author on appropriate request. Relevant parts of the data analysis pipeline are publicly available: https://github.com/fishpm/revival/.
