# Supplementary material for: Brain network changes and cognitive function after cardiac arrest
Source: Brain Commun. 2024 May 23;6(4):fcae174. doi: 10.1093/braincomms/fcae174 (PMC11264146; doi:10.1093/braincomms/fcae174)
Supplement: fcae174_Supplementary_Data [file fcae174_supplementary_data.zip › Original Submission.pdf]

OXFORD UNIVERSITY PRESS | Brain Communications

Brain network changes and cognitive function after cardiac arrest

Journal:	*Brain Communications*
Manuscript ID	BRAINCOM-2024-010
Manuscript Type:	Original Article
Date Submitted by the Author:	08-Jan-2024

Complete List of Authors:	Zarifkar, Pardis; Rigshospitalet Neurocentret, Department of Neurology<br>Wagner, Mette; Rigshospitalet, Department of Cardiology<br>Fisher, Patrick; Rigshospitalet Neurocentret, Neurobiology Research Unit; University of Copenhagen, Department of Drug Design and Pharmacology<br>Stenbæk, Dea; Rigshospitalet Neurocentret, Neurobiology Research Unit and Center for Integrated Molecular Brain Imaging; University of Copenhagen, Department of Clinical Medicine<br>Berg, Selina; Rigshospitalet, Cardiology; University of Copenhagen, Department of Clinical Medicine<br>Knudsen, Gitte; Rigshospitalet Neurocentret, Neurobiology Research Unit; University of Copenhagen, Department of Clinical Medicine<br>Benros, Michael E.; Copenhagen University Hospital, Copenhagen Research Centre for Biological and Precision Psychiatry, Mental Health Centre Copenhagen; University of Copenhagen, Department of Psychology<br>Kondziella, Daniel; Rigshospitalet Neurocentret, Department of Neurology; University of Copenhagen, Department of Clinical Medicine<br>Hassager, Christian; Rigshospitalet, Department of Cardiology; University of Copenhagen, Department of Clinical Medicine

Keywords:	Cardiac arrest, Cognitive dysfunction, Brain Mapping, Neural networks, Functional neuroimaging

SCHOLARONE™
Manuscripts

Title: Brain network changes and cognitive function after cardiac arrest

Running title: Brain networks after cardiac arrest

Authors: Pardis Zarifkar[1], Mette Kirstine Wagner[2], Patrick MacDonald Fisher[3,4], Dea Siggaard Stenbæk[4, 5], Selina Kikkenborg Berg[2,5], Gitte Moos Knudsen[4,5], Michael Eriksen Benros[6,7], Daniel Kondziella[1,5] and Christian Hassager[2,5]

Affiliations
[1]Department of Neurology, Copenhagen University Hospital, Rigshospitalet, Copenhagen, Denmark
[2]Department of Cardiology, Copenhagen University Hospital, Rigshospitalet, Copenhagen, Denmark
[3]Department of Drug Design and Pharmacology, University of Copenhagen
[4]Neurobiology Research Unit, Copenhagen University Hospital, Rigshospitalet, Copenhagen, Denmark
[5]Department of Clinical Medicine, University of Copenhagen, Copenhagen, Denmark
[6]Copenhagen Research Centre for Biological and Precision Psychiatry, Mental Health Centre Copenhagen, Copenhagen University Hospital, Copenhagen, Denmark
[7]Department of Psychology, Faculty of Social Sciences, University of Copenhagen, Copenhagen, Denmark

For Review Only

Tables: 2
Figures: 3
Supplemental tables: 1
Word Count, Abstract: 198
Word Count, Manuscript: 3401

Corresponding authors:

Christian Hassager, MD, DMSC
Department of Cardiology, Rigshospitalet, Copenhagen University Hopsital
Inge Lehmanns Vej 8, Copenhagen 2100, Denmark (christian.hassager@regionh.dk)

&

Daniel Kondziella, MD, Dr. philos.
Department of Neurology, Rigshospitalet, Copenhagen University Hospital
Inge Lehmanns Vej 8, Copenhagen 2100, Denmark (daniel.kondziella@regionh.dk)

Abstract

Survival rates after out-of-hospital cardiac arrest (OHCA) have improved over the past two decades. Despite this progress, long-term cognitive impairment remains prevalent even in those with early recovery of consciousness after OHCA; however, little is known about the determinants and underlying mechanisms. We utilized the REcovery after cardiac arrest surVIVAL (REVIVAL) cohort of OHCA survivors who fully regained consciousness to correlate cognition measurements with brain network changes using resting state functional MRI and the Montreal Cognitive Assessment (MoCA) at hospital discharge and a comprehensive neuropsychological assessment at three-month follow-up. About half of OHCA survivors displayed cognitive impairments at discharge, and in most, cognitive deficits persisted at three-month follow-up, particularly in the executive and visuospatial functions. Compared to healthy controls, OHCA survivors exhibited increased connectivity between resting-state networks, particularly involving the frontoparietal network. The increased connectivity between the frontoparietal and visual network was associated with less favorable cognitive outcomes (β=14.0, p=0.01), while higher education seemed to confer some cognitive protection (β=-2.06, p=0.03). In sum, the data highlight the importance of subtle cognitive impairment, also in OHCA survivors who are eligible for home discharge, and the potential of fMRI to identify alterations in brain networks correlating with cognitive outcomes.

Keywords: Cardiac arrest, cognitive dysfunction, brain mapping, neural networks, functional neuroimaging.

For Review Only

Introduction

Survival rates for out-of-hospital cardiac arrest (OHCA) have significantly improved in developed nations, showing a fourfold increase over the past two decades.[1] Europe and the United States report approximately 275,000[2] and 356,000 [3] OHCA cases annually, with about 10% of patients surviving until hospital discharge.[4] However, nearly half of these survivors suffer cognitive decline compared to pre-cardiac arrest, especially regarding memory, executive functions, and processing speed, which persists at least up to a year post-discharge.[5–9] The link between global ischemia and post-OHCA cognitive effects is well established,[10,11] yet cognitive dysfunction often goes undetected by standard clinical tools like the Cerebral Performance Category and Modified Rankin Scale.[4,12] This is particularly observed for survivors without visible structural brain injury on standard neuroimaging, including those eligible for home discharge.[4]

This study, which was part of the REcovery after cardiac arrest surVIVAL (REVIVAL) study,[13,14] investigated brain network changes using functional magnetic resonance imaging (fMRI) correlated with cognitive function in OHCA survivors who were well enough to be discharged home. We hypothesized that OHCA survivors would exhibit distinct cognitive profiles and brain connectivity patterns on fMRI, carrying prognostic implications also in the presence of unremarkable structural brain imaging. Our objectives were threefold: to assess cognitive function at discharge and again at three-month follow-up; to compare fMRI profiles between OHCA survivors and healthy controls; and to identify potential demographic, clinical, and neuroimaging predictors of cognitive trajectories.

Methods

For Review Only

Study design

The REVIVAL study at Copenhagen University Hospital, Rigshospitalet, is a prospective analysis focusing on OHCA survivors ready for home discharge. In a subset of these participants fMRI was performed.

Participant enrollment

From January 2018 to February 2022, first-time OHCA survivors of presumed cardiac origin as defined by the Utstein template, were enrolled.[15] The initial study protocol is available for reference.[13] Eligibility criteria included Danish language proficiency and written informed consent provided 4-9 days post analgesic-sedation withdrawal. Exclusions were based on MRI contraindications, premorbid neurological, cognitive, or psychiatric conditions, previous cerebrovascular or traumatic brain injuries, a high depression score (> 11) on Hospital Anxiety and Depression Scale,[16] and anticipated lack of participation to a three-month follow-up.

Prior to discharge, participants underwent cognitive assessments and neuroimaging. After three months, a comprehensive neuropsychological test battery was administered (see below). Comparative imaging data was included from 124 healthy individuals who underwent identical MRI scans at our institution, accessed via the Cimbi database.[17]

Clinical and cognitive assessments

Cardiac work-up was done according to standard clinical procedures including echocardiography and percutaneous coronary angiography where indicated. Delirium during admission was assessed using the 4AT scale, a rapid screening tool combining observational and interview-based elements to assess alertness, attention, fluctuations, and disorganized thinking.[18]

For Review Only

Functional status at discharge was assessed using three scales: i) Barthel Index-20[19]: this index quantifies the ability to perform ten basic activities of daily living, providing a measure of a patients independence and functional status, ii) Modified Rankin Scale[20]: this scale assesses the degree of disability or dependence in daily activities, and iii) Cerebral Performance Category Scale[21]: specifically used for cardiac arrest survivors, this scale classifies neurological outcomes ranging from good cerebral performance to death.

Cognitive function was assessed at discharge using the Montreal Cognitive Assessment (MoCA),[22] adjusted for educational level. A score of 26 or higher was considered indicative of normal cognitive function, while scores below 23 suggested cognitive impairment.[22] At three-month follow-up, participants' cognitive functions were reassessed by a health care professional blinded to the participants previous data. The neuropsychological test battery assessed episodic memory, executive function, visuospatial construction, and verbal fluency as detailed in **Table 1**. Cognitive status was classified as either favorable or unfavorable. We used a conservative criterion, defining clinically significant cognitive impairment as a score ≥1.5 standard deviations below the normative mean in two tests within the same cognitive domain, or in at least one test across two or more cognitive domains.[23–27]

For Review Only

Structural and functional MRI imaging and processing

MRI scans were performed using a Siemens MAGNETOM 3T Prisma scanner with a 64-channel head and neck coil. We acquired a high-resolution, whole-brain T1-weighted sequence (MP-RAGE) with the following parameters: inversion time of 900 ms, repetition time of 1900 ms, echo time of 2.58 ms, flip angle of 9°, in-plane matrix of 256 x 256 mm, in-plane resolution of 0.9 x 0.9 mm, and 224 slices (0.9 mm slice thickness). Resting-state fMRI (rs-fMRI) scans were acquired using a T2*-weighted gradient echo-planar imaging (EPI) sequence with a repetition time of 2000 ms, echo time of 30 ms, and flip angle of 90°, in-plane matrix of 64 x

64 mm, in-plane resolution of 3.6 x 3.6 mm, slice thickness of 3 mm with a 0.75 mm gap, 32 slices acquired interleaved, bottom-up. Individuals were scanned for 10 minutes, i.e., 300 whole-brain volumes were acquired. An accompanying field map was generated to correct spatial distortions in the EPI images. During the rs-fMRI scan, participants were instructed to keep their eyes closed and let their minds wander without falling asleep.

Preprocessing

Data were preprocessed using SPM12[1]and spmup toolbox[2] in Matlab R2021.[3] Steps included slice-timing correction, spatial realignment and unwarping, co-registration with T1-weighted structural images, tissue-type segmentation (based on T1-weighted structural images), spatial normalization into Montreal Neurological Institute space (final voxel size 3x3x3 mm) and smoothing with a 9 mm full-width at half-maximum kernel. Further denoising was performed with CONN v19.[28,29] The time series underwent bandpass filtering (0.008-0.09 Hz) and noise sources were regressed from the time series including anatomical component correction (aCompCor),[30] motion parameters, and outlier volumes, which were identified via Artifact Detection Tools.[31]

Data analysis

Functional Connectivity analysis was conducted using mean denoised time series extracted from regions of interest (ROIs) defined a priori by the “networks” atlas in CONN, which categorizes 32 regions of the brain into eight resting-state networks (default mode, dorsal attention, frontoparietal, language, salience, sensorimotor, visual, and cerebellar networks). Connectivity between ROI pairs was assessed using Fisher's r-to-z transformation of Pearson's

[1] https://www.fil.ion.ucl.ac.uk/spm/software/spm12
[2] https://github.com/CPernet/spmup
[3] https://www.mathworks.com

For Review Only

rho, i.e., z = artanh(r), where *r* represents the Pearson's rho correlation coefficient and *artanh* represents the inverse hyperbolic tangent function. For each scan session, a region-to-region connectivity matrix was compiled. Within-network connectivity was calculated as the mean connectivity across ROI pairs within the same network (i.e., eight within-network measures). Between-network connectivity referred to the mean connectivity across ROI pairs spanning different networks (i.e., 28 between-network pairs). Global functional connectivity was determined as the mean connectivity across all ROI pairs, irrespective of network affiliation.

Statistical analyses

Descriptive statistics were used to summarize demographic and clinical characteristics of OHCA survivors and healthy controls. Differences in functional connectivity were analyzed using Analysis of Covariance (ANCOVA), adjusted for age, sex, and education level (0- primary, 1- secondary, 2- tertiary). The Bonferroni correction was applied for multiple comparison adjustments. Within and between-network connectivity patterns were visualized using heatmaps. To identify predictive factors for cognitive function three months post-discharge, we employed Least Absolute Shrinkage and Selection Operator (LASSO) regression, selecting from a comprehensive set of demographic, clinical and neuroimaging variables. These included AED fibrillations, time to return of spontaneous circulation (ROSC; minutes), cardiac ejection fraction (%), targeted temperature management, sedation level (0- none, 1- propofol or remifentanil, 2- propofol or remifentanil and benzodiazepines), coma duration (hours), hospitalization length (days), delirium incidence, and MoCA score at discharge, along with global, within-, and between-network connectivity. The strength of regularization in LASSO was determined by the optimal lambda parameter, identified via cross-validation within the glmnet framework in R. Post-LASSO, regular logistic regression was conducted between binary cognitive outcomes (favorable/unfavorable) and the selected

For Review Only

variables. The logistic regression model was adjusted for demographic factors if not selected by LASSO in sensitivity analyses. All analyses were conducted using R statistical software v. 4.3.2 (R Core Team, Vienna, Austria, 2022).

Ethical approval

The study adhered to the Declaration of Helsinki and received approval from the regional Danish Research Ethics Committee (H-18046155).

Results

Demographic and clinical characteristics

Between January 2018 to February 2022, we identified 45 eligible OHCA survivors for the REVIVAL fMRI sub-study. Of these, data were not acquired in seven patients due to COVID-19 restrictions and one due to an incidental finding of metal splints, resulting in a final cohort of 37 participants. The participant selection process, including specific exclusion reasons, is detailed in the flowchart (**Figure 1**).

Demographic and clinical characteristic are detailed in **Table 2.** The median age of the final cohort was 53 years (IQR:20). The majority (n=31, 84%) were male, and eight (22%) had completed tertiary education. At the time of OHCA, 36 (97%) exhibited a shockable rhythm and achieved ROSC within a mean of 17±15 minutes. Bystander CPR was administered in 33 (89%) of cases. During ICU stay, 25 (67%) received sedation with propofol, remifentanil and/or benzodiazepines, 22 (59%) underwent targeted temperature management, and 5 (14%) experienced delirium. An implantable cardioverter defibrillator (ICD) was implanted in 35 (96%) of patients. Upon discharge, 36 (97%) of the patients were evaluated as having intact or

For Review Only

only mildly impaired neurological function, as indicated by scoring 1 on the Cerebral Performance Scale.

At three-month follow-up, 30 of the 37 OHCA patients were reassessed; median age 53 years (IQR: 19), 26 (87%) males, 8 (27%) with tertiary education. The control neuroimaging group comprised 124 healthy individuals, with a median age of 27 (IQR:8), 52 (42%) were male and 46 (37%) had tertiary education. The OHCA group was significantly older (mean age 51 vs. 30, t =-8.57, df =43.769, p<0.001) and showed differences in sex (χ^2=18.3, df=1, p<0.001) and education distributions (χ^2=40.0, df=2, p<0.001) compared to controls.

Cognitive outcomes and structural neuroimaging in OHCA survivors

At discharge, the mean MoCA score for the OHCA group was 25±3, with scores ranging from 18-29. Nine (24%) scored between 24 and 26 points, indicating possible cognitive impairment, and eight (22%) scored below 23, indicating definite cognitive impairment. No significant associations were found between MoCA scores and age (r=0.10, p=0.56), sex (t=-0.77, p=0.46), or education (F=1.21, p=0.31), suggesting that demographic factors did not have a major influence on cognitive performance as measured by MoCA in our sample.

At three-month follow-up, 15 (50%) OHCA survivors met criteria for unfavorable cognitive outcomes, with impairment in one or more cognitive domains. Specific impairment was observed in executive function (n=19, 32%), visuospatial abilities (n=7, 23%), verbal fluency (n=6, 20%), and episodic memory (n=5, 17%). There were no significant differences in age (t=-0.20, p=0.84), sex (X^2=0.06, p=0.80), or education (W=151, p=0.07) between cognitively favorable and unfavorable patient groups.

For Review Only

Resting-state fMRI findings

Global connectivity patterns

Initial analyses indicated higher global connectivity in OHCA survivors compared to healthy controls (**Figure 2A**; W=-2.98, p=0.004). After adjusting for demographics (age, sex, and education), this difference was not significant (**Figure 2B;** β=0.01, p=0.53). In the adjusted model, sex emerged as a significant factor, with males exhibiting lower global connectivity than females (β=0.03, p=0.01). Age and education were not significantly associated with global connectivity (age: β=0.001, p=0.13; education: β=-0.0004, p=0.10).

Within-network and between-network connectivity

There were no significant differences (p>0.05) in within-network between OHCA survivors and controls. However, between-network connectivity was higher in OHCA survivors, especially involving frontoparietal and default mode networks. After controlling for demographic variables and adjusting for multiple comparisons, significant increases persisted between the frontoparietal and visual networks (β=0.14, p=0.01), as well as the frontoparietal and sensorimotor networks (β=0.17, p=0.01; **Supplemental Table S1, Figure 3**).

Demographic, clinical and neuroimaging predictors of cognitive outcomes in OHCA survivors

Lasso regression was employed to select relevant variables from a broad set of demographic, clinical, and neuroimaging factors potentially influencing cognitive outcomes at follow-up. Significant variables identified were education, cardiac ejection fraction, MoCA score at discharge, along with connectivity between the frontoparietal and visual networks (henceforth referred to as frontoparietal-visual connectivity), and between the sensorimotor and language networks.

For Review Only

Subsequent logistic regression analysis confirmed the associations of education and frontoparietal-visual connectivity with favorable or unfavorable cognitive outcomes. Specifically, higher education was associated with a reduced risk of unfavorable cognitive outcomes (β=-2.06, p=0.03), whereas increased frontoparietal-visual connectivity was associated with a greater likelihood of unfavorable cognitive outcomes (β=14.0, p=0.01). Incorporating age and sex as covariates, the influence of education (β=-2.02, p=0.046) and frontoparietal-visual connectivity (β=13.8, $p < 0.01$) on cognitive outcomes remained significant. The inclusion of these covariates improved the model's fit, evidenced by a lower an Akaike Information Criterion (AIC) from 33.3 to 30.5.

Discussion

Cognitive function over time in OHCA survivors

Our study revealed significant cognitive impairment in OHCA survivors at discharge with 46% scoring below the normal threshold on the MoCA. At three-month follow-up, this impairment persisted, with 50% showing deficits in at least one cognitive domain. The most affected areas were executive and visuospatial functions, but deficits were also found in verbal fluency and episodic memory, highlighting the broad spectrum of cognitive challenges after OHCA. Notably, we found no significant correlation between immediate post-OHCA cognitive performance and demographic variables.

Studies on OHCA report a wide range of cognitive impairment, from 6% to 100% prevalence.[5–9,32–34] Such variability reflects methodological differences, diverse study populations, small sample sizes, and the retrospective nature of most studies.[6,35] Our findings align with those of similar prospective studies, where approximately half of OHCA survivors are found to be

For Review Only

cognitively impaired.[6,32–35] For instance, one study identified neuropsychological deficits in 24 out of 57 (42%) OHCA survivors, mainly in attention and motor skills;[34] however, the study's cognitive assessment was limited to the Trail Making Test, without separate measures for each cognitive domain. By contrast, a larger study with 184 OHCA survivors showed that 53 (29%) experienced impairment in at least two cognitive domains, particularly in executive function, memory, and processing speed, seven months post-event.[5] This echoes earlier findings, such as one study reporting cognitive impairments in 50% of 38 OHCA survivors after six months,[32] and another study observing such impairments in 60% of 57 survivors after three months, with deficits in memory and planning.[34] Together, these studies underscore the persistence of cognitive challenges in OHCA survivors, despite advances in clinical management and assessment techniques, and highlight the need for cognitive rehabilitation in post-OHCA care.

For Review Only

Functional reorganization after OHCA

Our study presents fMRI patterns in OHCA survivors well enough for home discharge, a cardiac population not previously explored with functional neuroimaging. Analyses revealed distinctive brain connectivity patterns between OHCA survivors at discharge and healthy controls. OHCA survivors had fMRI results compatible with increased global network connectivity, though this difference diminished when adjusted for demographic factors. In contrast, significant increases remained regarding connectivity between resting state networks, especially pertaining to the frontoparietal network. The latter network is important for cognitive control and the coordination of behavior, enabling rapid, accurate and flexible responses to goal-driven tasks,[36] and increased frontoparietal connectivity may indicate an inadequate compensatory response to altered brain networks, as observed in other cases of cerebral injury, such as traumatic brain injury or early neurodegenerative disease stages.[37–39] Unlike the

increases in between-network connectivity, within-network connectivity remained comparable to healthy controls, suggesting a resilience in core brain networks.

Demographic differences in age, sex, and education carry potential neurobiological implications for fMRI outcomes.[40–42] In our study, we observed an association between sex and network connectivity, with females exhibiting higher global connectivity than males. This finding is consistent with existing literature that documents both structural[43] and functional differences in brain connectivity between sexes.[44–50] Reports of functional differences, however, show greater diversity in their outcomes. For example, a study involving 336 females and 225 males reported higher local functional connectivity density in females.[44] Another study with 1685 participants from three cohorts, using resting-state connectivity for sex classification, identified distinct functional organization patterns in specific brain regions, but did not investigate global connectivity.[45] Changes in functional connectivity are also seen with age, with reports of a general decrease in global and within-network, especially involving cognitive and sensorimotor networks.[41] Previous research has shown that this age-related decrease is most marked in individuals aged 65 to 79 years, followed by an increase after 80 years.[40–42] This pattern suggests age-related connectivity changes and potential compensatory mechanisms, especially in between-network connectivity among older, more educated adults.[40–42,51] Given these findings, the younger, well-educated control group with a balanced sex distribution would be expected to have higher network connectivity. However, contrary to these expectations, it was the older, less educated, and predominantly male group of OHCA survivors who demonstrated increased network connectivity and heterogeneity. This deviation from brain network connectivity found in normal aging suggests that the increased network connectivity observed in our patients is a result of neuronal reorganization following OHCA, rather than being driven by demographic factors.

For Review Only

Demographic, clinical and neuroimaging predictors of cognitive function post-OHCA

Education, cardiac ejection fraction, MoCA scores at discharge, and frontoparietal-visual connectivity emerged as potential predictors of cognitive outcomes in OHCA survivors. Of these, education level and frontoparietal-visual connectivity were significantly associated with cognitive function at three-month follow-up. The correlation between higher education and more favorable cognitive outcomes is consistent with the cognitive reserve theory which posits that pre-existing cognitive abilities can mitigate the impact of brain injury,[52,53] and connectivity patterns associated with high cognitive reserve have been linked to better cognitive performance.[51] By contrast, increased frontoparietal-visual connectivity, possibly indicating an inadequate compensatory response,[37–39,51] was associated with poorer outcomes, suggesting that fMRI connectivity may have the potential to serve as a biomarker of cognitive function after OHCA.

While cardiac ejection fraction was related to cognitive outcomes, it was not a significant predictor in our study. This observation is consistent with previous research that associates very low ejection fractions with cognitive deficits.[54] In our cohort, ejection fraction was only moderately decreased, with only 4 (11%) performing under 40%, which may not have been low enough to impact cognitive function. Finally, the MoCA scores at discharge were only loosely correlated with later cognitive outcomes, potentially due to their limited sensitivity in detecting subtle, domain-specific deficits.[55,56] This finding emphasizes the importance of routine cognitive monitoring post-OHCA using comprehensive neuropsychological test batteries. Hence, OHCA survivors and their families and caregivers should be vigilant about potential delayed cognitive challenges, even if initial screening assessments are normal.

For Review Only

Strengths and limitations

Our study was novel in its focus on neural connectivity and cognitive outcomes in post-OHCA survivors without overt structural brain injury who were ready for home discharge. However, several limitations must be acknowledged. First, due to logistical reasons, neuroimaging and MoCA were not repeated at three-month follow-up. This precluded a direct comparison of brain network changes and cognitive function over time, a gap that future studies should address. Additionally, our study evaluated only resting-state functional connectivity. Cognition-related task-based fMRI may possibly yield more sensitive results in identifying prognostic brain imaging biomarkers, although this remains to be shown. Second, a more comprehensive assessment at discharge could have revealed domain-specific cognitive impairment in the acute stage. Additionally, a proper psychiatric assessment may have identified mood disorders known to be associated with cognitive deficits.[57] Third, our sample comprised 37 OHCA survivors enrolled consecutively over nearly five years. Although our sample is both demographically and clinically representative of a general OHCA population and larger than many similar cohorts,[3,4,58–61] it is unlikely to fully represent the entire spectrum of post-OCHA survivors without overt brain injury. Fourth, the pre-OHCA cognitive function of individuals could not be considered due to the inherent unpredictability of the cardiac event. Finally, the control group of 124 healthy participants was not demographically matched to the OHCA cohort. Although we adjusted for age, sex, and educational background, this approach has its limitations as already discussed. The cognitive reserve theory posits that while certain predictors indicate potential cognitive decline, actual outcomes are influenced by mitigating factors like education, intelligence, and neural plasticity.[62] Our study considered some of these elements, but future investigations should include larger groups and additional influencing factors. In sum, although our study does not fully capture the disparities in individual patient

For Review Only

performances, we believe it nevertheless adds insights into cognitive trajectories post-OHCA by integrating functional imaging, demographic, and clinical perspectives.

Conclusions

OHCA survivors with early recovery of consciousness and no visible structural brain injury can still exhibit substantial cognitive impairment which correlates with alterations in brain network connectivity, specifically increased between-network resting-state connectivity. In almost half of OHCA survivors, cognitive impairment persisted from hospital discharge to three-month follow-up, particularly affecting executive and visuospatial functions. Higher education seemed to confer some cognitive protection, whereas increased connectivity between the frontoparietal and visual network was associated with less favorable cognitive outcomes. These observations support the cognitive reserve theory and identify a potential fMRI biomarker for predicting post-OHCA cognitive trajectories.

For Review Only

Funding

This project was supported by The Research Fund of Rigshospitalet – Copenhagen University Hospital (E-22281-05), the Research Fund between Copenhagen University Hospital, Rigshospitalet and Odense University Hospital (R38-2015), The Danish Health Foundation (18-B-0235), Lundbeck Foundation (R349-2020-658, R268-2016-3925), and Novo Nordisk Foundation (NNF21OC0067769).

Declaration of interest: The authors have no relevant conflicts of interest related to the content of this study.

Data sharing: Anonymized data and protocols are available from the corresponding authors on appropriate requests.

Role of the funder: The funder had no influence in the study design, collection, analysis, or interpretation of data.

For Review Only

References

1. Yan S, Gan Y, Jiang N, et al. The global survival rate among adult out-of-hospital cardiac arrest patients who received cardiopulmonary resuscitation: a systematic review and meta-analysis. *Crit Care*. 2020;24(1). doi:10.1186/S13054-020-2773-2
2. Atwood C, Eisenberg MS, Herlitz J, Rea TD. Incidence of EMS-treated out-of-hospital cardiac arrest in Europe. *Resuscitation*. 2005;67(1):75-80. doi:10.1016/J.RESUSCITATION.2005.03.021
3. Benjamin EJ, Virani SS, Callaway CW, et al. Heart Disease and Stroke Statistics-2018 Update: A Report From the American Heart Association. *Circulation*. 2018;137(12):E67-E492. doi:10.1161/CIR.0000000000000558
4. Hagberg G, Ihle-Hansen H, Sandset EC, Jacobsen D, Wimmer H, Ihle-Hansen H. Long Term Cognitive Function After Cardiac Arrest: A Mini-Review. *Front Aging Neurosci*. 2022;14. doi:10.3389/FNAGI.2022.885226
5. Blennow Nordström E, Lilja G, Vestberg S, et al. Neuropsychological outcome after cardiac arrest: A prospective case control sub-study of the Targeted hypothermia versus targeted normothermia after out-of-hospital cardiac arrest trial (TTM2). *BMC Cardiovasc Disord*. 2020;20(1):1-11. doi:10.1186/S12872-020-01721-9/TABLES/4
6. Byron-Alhassan A, Collins B, Bedard M, et al. Cognitive dysfunction after out-of-hospital cardiac arrest: Rate of impairment and clinical predictors. *Resuscitation*. 2021;165:154-160. doi:10.1016/J.RESUSCITATION.2021.05.002
7. Moulaert VRMP, Verbunt JA, van Heugten CM, Wade DT. Cognitive impairments in survivors of out-of-hospital cardiac arrest: A systematic review. *Resuscitation*. 2009;80(3):297-305. doi:10.1016/J.RESUSCITATION.2008.10.034
8. Moulaert VRM, Van Heugten CM, Winkens B, et al. Early neurologically-focused follow-up after cardiac arrest improves quality of life at one year: A randomised controlled trial. *Int J Cardiol*. 2015;193:8-16. doi:10.1016/J.IJCARD.2015.04.229
9. Zook N, Voss S, Blennow Nordström E, et al. Neurocognitive function following out-of-hospital cardiac arrest: A systematic review. *Resuscitation*. 2022;170:238-246. doi:10.1016/J.RESUSCITATION.2021.10.005
10. Horstmann A, Frisch S, Jentzsch RT, Müller K, Villringer A, Schroeter ML. Resuscitating the heart but losing the brain: brain atrophy in the aftermath of cardiac arrest. *Neurology*. 2010;74(4):306-312. doi:10.1212/WNL.0B013E3181CBCD6F
11. Iadecola C. The Neurovascular Unit Coming of Age: A Journey through Neurovascular Coupling in Health and Disease. *Neuron*. 2017;96(1):17-42. doi:10.1016/J.NEURON.2017.07.030
12. Pek PP, Fan KC, Ong MEH, et al. Determinants of health-related quality of life after out-of-hospital cardiac arrest (OHCA): A systematic review. *Resuscitation*. 2023;188:109794. doi:10.1016/J.RESUSCITATION.2023.109794
13. Wagner MK, Berg SK, Hassager C, et al. Cognitive impairment and psychopathology in out-of-hospital cardiac arrest survivors in Denmark: The REVIVAL cohort study protocol. *BMJ Open*. 2020;10(9). doi:10.1136/BMJOPEN-2020-038633
14. Wagner MK, Berg SK, Hassager C, et al. Cognitive impairment and psychopathology in sudden out-of-hospital cardiac arrest survivors: Results from the REVIVAL cohort study. *Resuscitation*. 2023;192. doi:10.1016/J.RESUSCITATION.2023.109984
15. Nolan JP, Berg RA, Andersen LW, et al. Cardiac Arrest and Cardiopulmonary Resuscitation Outcome Reports: Update of the Utstein Resuscitation Registry Template for In-Hospital Cardiac Arrest: A Consensus Report From a Task Force of the International Liaison Committee on Resuscitation (American Heart Association, European Resuscitation Council, Australian and New Zealand Council on Resuscitation, Heart and Stroke Foundation of Canada, InterAmerican Heart

For Review Only

Foundation, Resuscitation Council of Southern Africa, Resuscitation Council of Asi. *Circulation*. 2019;140(18):e746-e757. doi:10.1161/CIR.0000000000000710
16. Zigmond AS, Snaith RP. The hospital anxiety and depression scale. *Acta Psychiatr Scand*. 1983;67(6):361-370. doi:10.1111/J.1600-0447.1983.TB09716.X
17. Knudsen GM, Jensen PS, Erritzoe D, et al. The Center for Integrated Molecular Brain Imaging (Cimbi) database. *Neuroimage*. 2016;124(Pt B):1213-1219. doi:10.1016/J.NEUROIMAGE.2015.04.025
18. Bellelli G, Morandi A, Davis DHJ, et al. Validation of the 4AT, a new instrument for rapid delirium screening: a study in 234 hospitalised older people. *Age Ageing*. 2014;43(4):496-502. doi:10.1093/AGEING/AFU021
19. Collin C, Wade DT, Davies S, Horne V. The Barthel ADL Index: a reliability study. *Int Disabil Stud*. 1988;10(2):61-63. doi:10.3109/09638288809164103
20. Wilson JTL, Hareendran A, Grant M, et al. Improving the assessment of outcomes in stroke: use of a structured interview to assign grades on the modified Rankin Scale. *Stroke*. 2002;33(9):2243-2246. doi:10.1161/01.STR.0000027437.22450.BD
21. Grenvik Ake, Safar P. Brain failure and resuscitation. Published online 1981:268. Accessed December 15, 2023. https://books.google.com/books/about/Brain_Failure_and_Resuscitation.html?id=xJ9rAAAAMAAJ
22. Nasreddine ZS, Phillips NA, Bédirian V, et al. The Montreal Cognitive Assessment, MoCA: a brief screening tool for mild cognitive impairment. *J Am Geriatr Soc*. 2005;53(4):695-699. doi:10.1111/J.1532-5415.2005.53221.X
23. Jak AJ, Bondi MW, Delano-Wood L, et al. Quantification of five neuropsychological approaches to defining mild cognitive impairment. *Am J Geriatr Psychiatry*. 2009;17(5):368. doi:10.1097/JGP.0B013E31819431D5
24. Schinka JA, Loewenstein DA, Raj A, et al. Defining Mild Cognitive Impairment: Impact of Varying Decision Criteria on Neuropsychological Diagnostic Frequencies and Correlates. *Am J Geriatr Psychiatry*. 2010;18(8):684. doi:10.1097/JGP.0B013E3181E56D5A
25. Robertson K, Larson EB, Crane PK, et al. Using Varying Diagnostic Criteria to Examine Mild Cognitive Impairment Prevalence and Predict Dementia Incidence in a Community-Based Sample. *J Alzheimers Dis*. 2019;68(4):1439-1451. doi:10.3233/JAD-180746
26. Wong CG, Thomas KR, Edmonds EC, et al. Neuropsychological Criteria for Mild Cognitive Impairment in the Framingham Heart Study's Old-Old. *Dement Geriatr Cogn Disord*. 2018;46(5-6):253-265. doi:10.1159/000493541
27. Zarifkar P, Kim J, La C, et al. Cognitive impairment in Parkinson's Disease is associated with Default Mode Network subsystem connectivity and cerebrospinal fluid Aβ. *Parkinsonism Relat Disord*. 2021;83:71. doi:10.1016/J.PARKRELDIS.2021.01.002
28. CONN Toolbox. https://web.conn-toolbox.org/
29. Whitfield-Gabrieli S, Nieto-Castanon A. Conn: a functional connectivity toolbox for correlated and anticorrelated brain networks. *Brain Connect*. 2012;2(3):125-141. doi:10.1089/BRAIN.2012.0073
30. Behzadi Y, Restom K, Liau J, Liu TT. A component based noise correction method (CompCor) for BOLD and perfusion based fMRI. *Neuroimage*. 2007;37(1):90-101. doi:10.1016/J.NEUROIMAGE.2007.04.042
31. Artifact Detection Tools. http://web.mit.edu/swg/software.htm

For Review Only

32. Sauvé MJ, Dolittle N, Walker AJ, Paul SM SM. Factors associated with cognitive recovery after cardiopulmonary resuscitation. *American Journal of Critical Care*. 1996;5(2):127-139.
33. Roine RO, S Kajaste MK. Neuropsychological sequelae of cardiac arrest. *JAMA*. 1993;262(2):237-242.
34. Van Alem AP, De Vos R, Schmand B, Koster RW. Cognitive impairment in survivors of out-of-hospital cardiac arrest. *Am Heart J*. 2004;148(3):416-421. doi:10.1016/j.ahj.2004.01.031
35. Moulaert VRMP, Verbunt JA, van Heugten CM, Wade DT. Cognitive impairments in survivors of out-of-hospital cardiac arrest: a systematic review. *Resuscitation*. 2009;80(3):297-305. doi:10.1016/J.RESUSCITATION.2008.10.034
36. Marek S, Dosenbach NUF. The frontoparietal network: function, electrophysiology, and importance of individual precision mapping. *Dialogues Clin Neurosci*. 2018;20(2):133-141. doi:10.31887/DCNS.2018.20.2/SMAREK
37. Hammeke TA, McCrea M, Coats SM, et al. Acute and Subacute Changes in Neural Activation during the Recovery from Sport-Related Concussion. *Journal of the International Neuropsychological Society*. 2013;19(8):863-872. doi:10.1017/S1355617713000702
38. Farràs-Permanyer L, Guàrdia-Olmos J, Peró-Cebollero M. Mild cognitive impairment and fMRI studies of brain functional connectivity: the state of the art. *Front Psychol*. 2015;6:1095. doi:10.3389/FPSYG.2015.01095
39. Audoin B, Ibarrola D, Ranjeva JP, et al. Compensatory cortical activation observed by fMRI during a cognitive task at the earliest stage of MS. *Hum Brain Mapp*. 2003;20(2):51-58. doi:10.1002/HBM.10128
40. Zhang C, Dougherty CC, Baum SA, White T, Michael AM. Functional connectivity predicts gender: Evidence for gender differences in resting brain connectivity. *Hum Brain Mapp*. 2018;39(4):1765. doi:10.1002/HBM.23950
41. Betzel RF, Byrge L, He Y, Goñi J, Zuo XN, Sporns O. Changes in structural and functional connectivity among resting-state networks across the human lifespan. *Neuroimage*. 2014;102(P2):345-357. doi:10.1016/J.NEUROIMAGE.2014.07.067
42. Farras-Permanyer L, Mancho-Fora N, Montalà-Flaquer M, et al. Age-related changes in resting-state functional connectivity in older adults. *Neural Regen Res*. 2019;14(9):1544. doi:10.4103/1673-5374.255976
43. Ritchie SJ, Cox SR, Shen X, et al. Sex Differences in the Adult Human Brain: Evidence from 5216 UK Biobank Participants. *Cereb Cortex*. 2018;28(8):2959-2975. doi:10.1093/CERCOR/BHY109
44. Tomasi D, Volkow ND. Gender differences in brain functional connectivity density. *Hum Brain Mapp*. 2012;33(4):849. doi:10.1002/HBM.21252
45. Weis S, Patil KR, Hoffstaedter F, Nostro A, Yeo BTT, Eickhoff SB. Sex Classification by Resting State Brain Connectivity. *Cerebral Cortex*. 2020;30(2):824-835. doi:10.1093/CERCOR/BHZ129
46. Ingalhalikar M, Smith A, Parker D, et al. Sex differences in the structural connectome of the human brain. *Proc Natl Acad Sci U S A*. 2014;111(2):823-828. doi:10.1073/PNAS.1316909110/ASSET/1CF26281-5D58-402C-8CC1-523603E1BD4C/ASSETS/GRAPHIC/PNAS.1316909110I6.GIF
47. Satterthwaite TD, Wolf DH, Roalf DR, et al. Linked Sex Differences in Cognition and Functional Connectivity in Youth. *Cerebral Cortex*. 2015;25(9):2383-2394. doi:10.1093/CERCOR/BHU036

For Review Only

48. Gaillard A, Fehring DJ, Rossell SL. Sex differences in executive control: A systematic review of functional neuroimaging studies. *Eur J Neurosci*. 2021;53(8):2592-2611. doi:10.1111/EJN.15107
49. Alfano V, Cavaliere C, Di Cecca A, et al. Sex differences in functional brain networks involved in interoception: An fMRI study. *Front Neurosci*. 2023;17. doi:10.3389/FNINS.2023.1130025
50. Xu M, Liang X, Ou J, Li H, Luo YJ, Tan LH. Sex Differences in Functional Brain Networks for Language. *Cereb Cortex*. 2020;30(3):1528-1537. doi:10.1093/CERCOR/BHZ184
51. Varela-López B, Cruz-Gómez ÁJ, Lojo-Seoane C, et al. Cognitive reserve, neurocognitive performance, and high-order resting-state networks in cognitively unimpaired aging. *Neurobiol Aging*. 2022;117:151-164. doi:10.1016/J.NEUROBIOLAGING.2022.05.012
52. Stern Y. Cognitive reserve in ageing and Alzheimer's disease. *Lancet neurology*. 2012;11(11):1006. doi:10.1016/S1474-4422(12)70191-6
53. Schneider EB, Sur S, Raymont V, et al. Functional recovery after moderate/severe traumatic brain injury: A role for cognitive reserve? *Neurology*. 2014;82(18):1636. doi:10.1212/WNL.0000000000000379
54. Goh FQ, Kong WKF, Wong RCC, et al. Cognitive Impairment in Heart Failure—A Review. *Biology (Basel)*. 2022;11(2):179. doi:10.3390/BIOLOGY11020179
55. Moafmashhadi P, Koski L. Limitations for Interpreting Failure on Individual Subtests of the Montreal Cognitive Assessment. *http://dx.doi.org/101177/0891988712473802*. 2013;26(1):19-28. doi:10.1177/0891988712473802
56. Coen RF, Robertson DA, Kenny RA, King-Kallimanis BL. Strengths and Limitations of the MoCA for Assessing Cognitive Functioning: Findings From a Large Representative Sample of Irish Older Adults. *J Geriatr Psychiatry Neurol*. 2016;29(1):18-24. doi:10.1177/0891988715598236
57. Marvel CL, Paradiso S. Cognitive and neurological impairment in mood disorders. *Psychiatr Clin North Am*. 2004;27(1):19. doi:10.1016/S0193-953X(03)00106-0
58. Navab E, Esmaeili M, Poorkhorshidi N, Salimi R, Khazaei A, Moghimbeigi A. Predictors of Out of Hospital Cardiac Arrest Outcomes in Pre-Hospital Settings; a Retrospective Cross-sectional Study. *Arch Acad Emerg Med*. 2019;7(1):e36. doi:10.22037/aaem.v7i1.370
59. Kotini-Shah P, Del Rios M, Khosla S, et al. SEX DIFFERENCES IN OUTCOMES FOR OUT-OF-HOSPITAL CARDIAC ARREST IN THE UNITED STATES. *Resuscitation*. 2021;163:6. doi:10.1016/J.RESUSCITATION.2021.03.020
60. Goldberger ZD, Chan PS, Berg RA, et al. Duration of resuscitation efforts and subsequent survival after in-hospital cardiac arrest. *Lancet*. 2012;380(9852):1473. doi:10.1016/S0140-6736(12)60862-9
61. Byron-Alhassan A, Collins B, Bedard M, et al. Cognitive dysfunction after out-of-hospital cardiac arrest: Rate of impairment and clinical predictors. *Resuscitation*. 2021;165:154-160. doi:10.1016/J.RESUSCITATION.2021.05.002
62. Buckner RL. Memory and Executive Function in Aging and AD: Multiple Factors that Cause Decline and Reserve Factors that Compensate. *Neuron*. 2004;44(1):195-208. doi:10.1016/J.NEURON.2004.09.006
63. Jensen CG, Hjordt L V., Stenbæk DS, et al. Development and psychometric validation of the verbal affective memory test. *Memory*. 2016;24(9):1208-1223. doi:10.1080/09658211.2015.1087573

For Review Only

64. Fine EM, Delis DC. Delis–Kaplan Executive Functioning System. *Encyclopedia of Clinical Neuropsychology*. Published online 2011:796-801. doi:10.1007/978-0-387-79948-3_1539

65. Wechsler, D. (1997) Wechsler Adult Intelligence Scale. 3rd Edition, The Psychological Corporation, San Antonio. - References - Scientific Research Publishing.

66. Stern RA, Singer EA, Duke LM, et al. The Boston Qualitative Scoring System for the Rey-Osterrieth Complex Figure: Description and interrater reliability. *Clinical Neuropsychologist*. 1994;8(3):309-322. doi:10.1080/13854049408404137

For Review Only

Tables

Table 1. **Neuropsychological test battery at three-month follow-up.**

This table details the neuropsychological test battery used to evaluate cognitive functions in OHCA survivors at three-month follow-up. The battery assesses key cognitive domains including episodic memory, executive function, verbal fluency, and visuospatial construction. Specific tests and measures used for each cognitive domain are listed, providing a detailed overview of the assessment methodology.

For Review Only

Cognitive domain	Test and primary cognitive outcome	Description
**Episodic memory**	**Verbal Affective Memory Task-26 (VMT-26):**[63] Condition A7	This test evaluates the recall of emotionally charged words. Condition A7 challenges participants to remember affective words 30 minutes after learning, following an intervening task with neutral words.
**Executive function**	**Delis-Kaplan Executive System (D-KEFS):** [64]	A set of neuropsychological tests designed to assess executive functions.
	**D-KEFS Color-Word Interference Test**: Condition 4 (switching)	The test involves reading words printed in incongruent colors and requires switching between naming the word and the color. Response time and accuracy are measured.
	**D-KEFS Design Fluency Test**:	Participants create unique designs by alternating between filled and empty dots. Completion time is measured.

	Condition 3 (Switching)	
	**D-KEFS Trail Making Test:** Condition 4 (switching)	Participants alternate between connecting numbered and lettered circles in sequence (e.g., 1 to A, A to 2). Completion time is measured.
	**D-KEFS Verbal Fluency Test**: Condition 3 (switching)	The test involves rapidly generating words based on alternating categories. Focuses on the ability to switch between categories and fluency of response.
	**Wechsler Adult Intelligence Scale (WAIS-IV):**[65] Letter-number sequencing	The test involves reordering mixed numbers and letters into ascending numerical and alphabetical order. Outcome is measured by accuracy and the ability to recall increasingly complex sequences.
**Verbal fluency**	**D-KEFS Verbal Fluency Test**: Condition 1 and 2	The test involves generating words beginning with a specific letter (Condition 1) and within a certain category (Condition 2). It focuses on lexical retrieval and category fluency.
**Visuospatial construction**	**Rey's Complex Figure Test and Recognition Trial:** [66] Conditions 3 (delayed recall) + 4 (recognition)	Condition 3 (Delayed Recall) involves reproducing a complex figure from memory after a delay. Condition 4 (Recognition) requires identifying the original figure from a set of figures.

For Review Only

Table 2. Demographic and clinical characteristics of OHCA survivors at discharge

**Demographic**	
**N = 37**	
**Age**	51±14; 53 (IQR:20)
**Sex (M)**	31 (84%)
**Education**	Primary: 20 (54%)<br>Secondary:7 (19%)<br>Tertiary: 8 (22%)<br>Unknown: 2 (5%)
**Employment status**	Full-time: 25 (68%)<br>Maternity, medical leave or part-time: 3 (8%)<br>Retired: 7 (19%)<br>Unknown: 1 (3%)
**Clinical**	
**Cardiovascular and neurological co-morbidities or**	Hypertension, COPD or CKD: 15 (41%)<br>IHD or AMI: 6 (16%)<br>Migraine: 1 (3%)
**Past cardiovascular interventions**	Percutaneous Coronary Intervention: 3 (8%)<br>Coronary Artery Bypass Graft: 1 (3%)
**Ejection fraction (%)**	52±10; 55 (IQR:10)
**Awake at arrival**	12 (32%)
**Shockable rhythm**	36 (97%)
**OHCA to ROSC (minutes)**	17±15; 10 (IQR:8)
**Coma (hours)**	26 (IQR: 45)
**ICU (hours)**	53 (IQR: 84)
**Hospital (days)**	12 (IQR:6)
**Delirium in the ICU**	5 (14%)
**Treatment**	
**Bystander CPR**	33 (89%)
**Initial rhythm**	Ventricular fibrillation: 33 (81%)<br>Pulseless electric activity: 4 (11%)<br>Unknown: 3 (8%)
**No. of AED fibrillations**	2±2; 2 (IQR:2)
**Sedation**	None: 12 (32%)<br>Propofol or remifentanil: 22 (59%)<br>Additionally, Benzodiazepines: 3 (8%)
**Targeted Temperature Management**	22 (59%)
**ICD Implantation**	35 (96%)
**Functional status at discharge**	
**Barthel Index**	100
**Modified Rankin Scale**	Score 1: 35 (95%)
**Cerebral Performance**	Score 1: 36 (97%)
**MoCA**	25±3; 26 (IQR: 4)

Data is presented either as mean ± standard deviation, median and interquartile range (IQR), or as a frequency and percentage (%).

For Review Only

Abbreviations: **AMI**, Acute Myocardial Infarction; **IHD**, Ischemic Heart Disease; **CKD**, Chronic Kidney Disease; **COPD**, Chronic Obstructive Pulmonary Disorder; **CPR,** Cardiopulmonary resuscitation; **M,** Male; ICD: Implantable Cardioverter Defibrillator; **MoCA**, Montreal Cognitive Assessment; **OHCA**, Out of Hospital Cardiac Arrest; **ROSC**, Return of Spontaneous Circulation

For Review Only

Figures

Figure 1. REVIVAL study participant inclusion flowchart.

This flowchart illustrates the participant selection process for the REVIVAL study, conducted from January 2018 to February 2022. Starting with 665 OHCA survivors, 37 were ultimately included in this sub-study, 30 of whom participated in the three-month follow-up. Details of the exclusion criteria are provided within the chart.

Abbreviations: **COVID-19,** Corona Virus Disease 2019; **OHCA**, Out of Hospital Cardiac Arrest.

Figure 2. Global connectivity after out-of-hospital-cardiac arrest.

- **A.** Boxplot of Fisher's z-transformed correlation coefficients comparing resting-state global connectivity between out-of-hospital cardiac arrest patients (n=37) and healthy controls (n=124).
- **B.** Boxplot of the residuals for global connectivity, adjusted for age, sex, and education, demonstrates diminished differences between the groups.

The whiskers represent the full range of the data, while the boxes show the interquartile range. The horizontal line within each box indicates the median value.

Abbreviations: **HC,** healthy controls; **PT**, patients.

Figure 3. Resting connectivity in OHCA survivors compared to healthy controls

- **A)** Heatmap illustrating Fisher's z-transformed correlation coefficients within and between resting state networks in OHCA survivors (n=37).

For Review Only

B) A comparative heatmap shows the differences in network connectivity between OHCA survivors and healthy controls. OHCA survivors exhibit increased between-network connectivity, especially involving the frontoparietal and default mode networks. This pattern suggests a potential compensatory response to maintain cognitive function in the OHCA survivor group.

Asterisks (*) denotes significantly altered connectivities after adjustment for multiple testing ($p<0.015$).

For Review Only

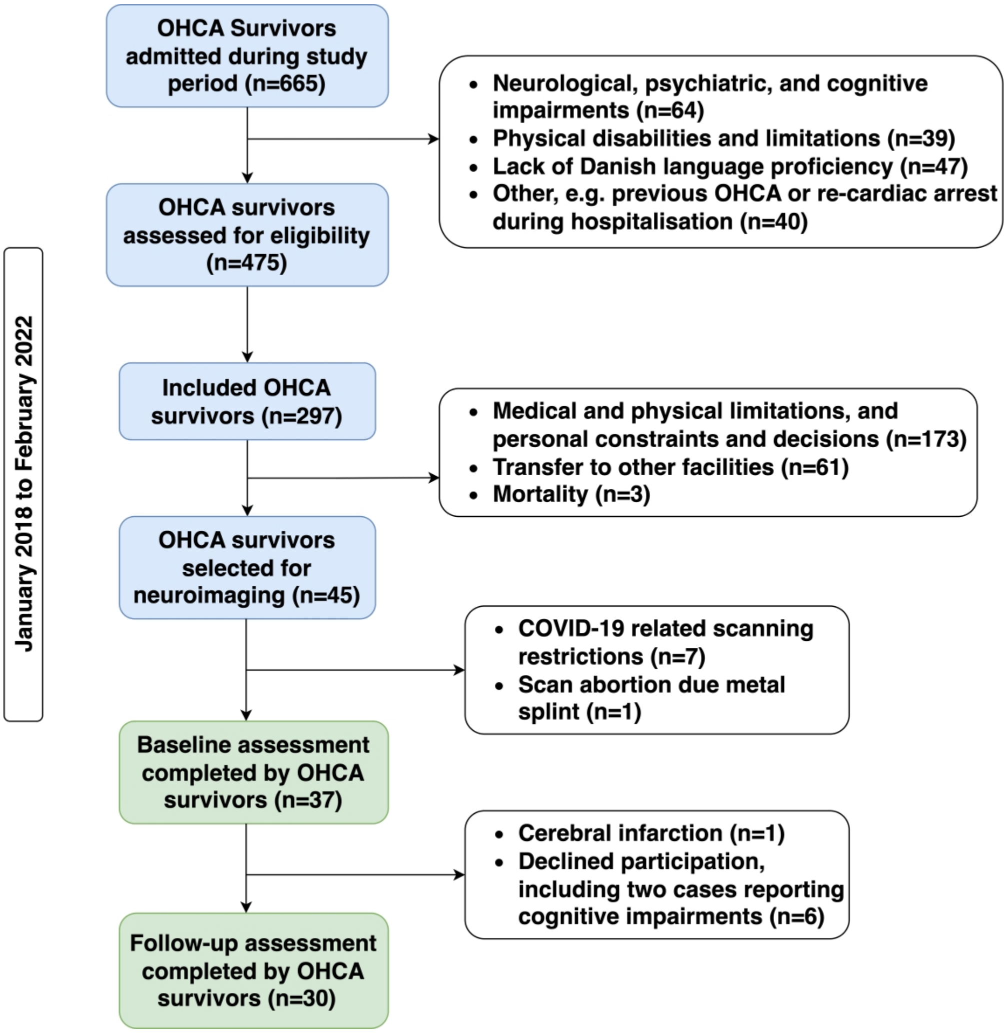

Figure 1. REVIVAL study participant inclusion flowchart. This flowchart illustrates the participant selection process for the REVIVAL study, conducted from January 2018 to February 2022. Starting with 665 OHCA survivors, 37 were ultimately included in this sub-study, 30 of whom participated in the three-month follow-up. Details of the exclusion criteria are provided within the chart. Abbreviations: COVID-19, Corona Virus Disease 2019; OHCA, Out of Hospital Cardiac Arrest.

504x517mm (236 x 236 DPI)

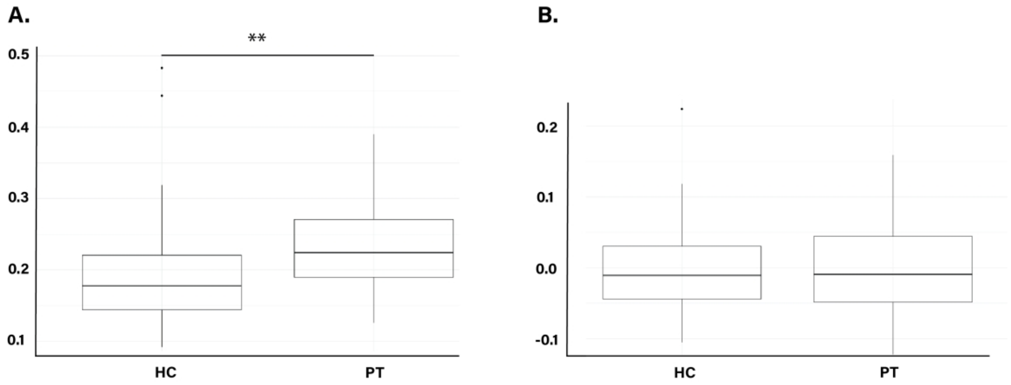

Figure 2. Global connectivity after out-of-hospital-cardiac arrest. A. Boxplot of Fisher's z-transformed correlation coefficients comparing resting-state global connectivity between out-of-hospital cardiac arrest patients (n=37) and healthy controls (n=124). B. Boxplot of the residuals for global connectivity, adjusted for age, sex, and education, demonstrates diminished differences between the groups.The whiskers represent the full range of the data, while the boxes show the interquartile range. The horizontal line within each box indicates the median value.Abbreviations: HC, healthy controls; PT, patients.

707x267mm (118 x 118 DPI)

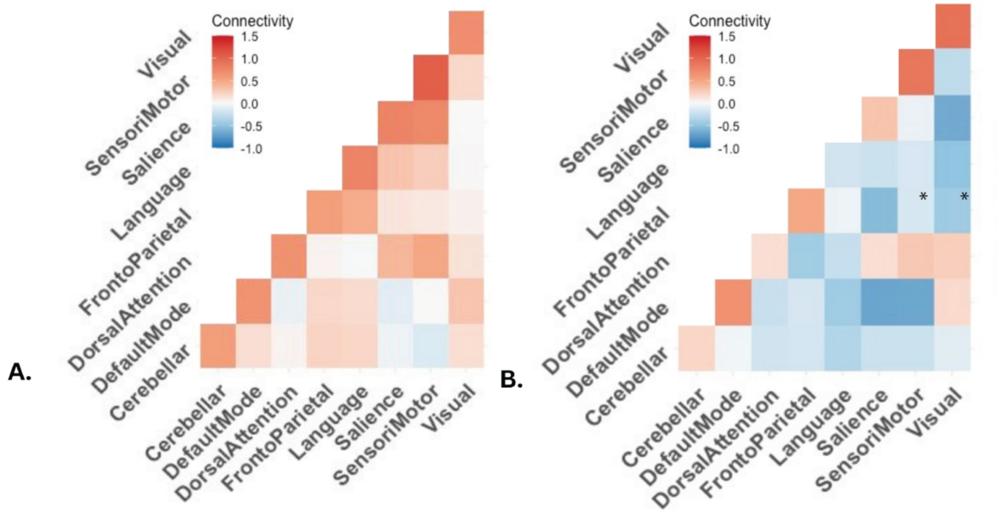

Figure 3. Resting connectivity in OHCA survivors compared to healthy controls. A. Heatmap illustrating Fisher's z-transformed correlation coefficients within and between resting state networks in OHCA survivors (n=37). B. A comparative heatmap shows the differences in network connectivity between OHCA survivors and healthy controls. OHCA survivors exhibit increased between-network connectivity, especially involving the frontoparietal and default mode networks. This pattern suggests a potential compensatory response to maintain cognitive function in the OHCA survivor group. Asterisks (*) denotes significantly altered connectivities after adjustment for multiple testing (p<0.015).

733x397mm (118 x 118 DPI)

Supplemental Table S1: Resting-state connectivity differences between OHCA survivors and healthy controls.

This table details differences in within- and between-network connectivity between OHCA survivors and healthy controls, accounting for demographic variables (age, sex, and education). After adjusting for multiple comparisons, significant increases persisted between the frontoparietal and visual networks, as well as the frontoparietal and sensorimotor networks. Bolded rows depict significant ($p<0.05$) or near-significant differences.

For Review Only

Network1_Network2	Estimate	Standard Error	Statistic	P-value	Adjusted P-Value
Cerebellar_Cerebellar	-0.1	0.082	-1.23	0.221	1
DefaultMode_Cerebellar	-0.014	0.039	-0.367	0.715	1
DorsalAttention_Cerebellar	-0.009	0.041	-0.232	0.817	1
**FrontoParietal_Cerebellar**	**-0.082**	**0.046**	**-1.778**	**0.078**	**1**
Language_Cerebellar	-0.043	0.039	-1.098	0.274	1
Salience_Cerebellar	0	0.04	-0.012	0.99	1
SensoriMotor_Cerebellar	0.066	0.046	1.417	0.159	1
Visual_Cerebellar	0.048	0.048	0.984	0.327	1
**DefaultMode_DefaultMode**	**0.071**	**0.04**	**1.772**	**0.079**	**1**
DorsalAttention_DefaultMode	0.002	0.045	0.038	0.969	1
FrontoParietal_DefaultMode	-0.024	0.037	-0.645	0.52	1
Language_DefaultMode	0.037	0.048	0.76	0.449	1
Salience_DefaultMode	-0.025	0.049	-0.514	0.608	1
SensoriMotor_DefaultMode	0.066	0.055	1.214	0.227	1
Visual_DefaultMode	0.067	0.049	1.362	0.176	1
DorsalAttention_DorsalAttention	-0.022	0.056	-0.391	0.697	1
FrontoParietal_DorsalAttention	0.054	0.039	1.382	0.17	1
Language_DorsalAttention	0.036	0.05	0.719	0.474	1
Salience_DorsalAttention	0.007	0.037	0.185	0.853	1
SensoriMotor_DorsalAttention	0.008	0.055	0.14	0.889	1
Visual_DorsalAttention	-0.017	0.048	-0.35	0.727	1
FrontoParietal_FrontoParietal	**-0.098**	**0.05**	**-1.944**	**0.054**	**1**
Language_FrontoParietal	-0.022	0.041	-0.546	0.586	1

For Review Only

Salience_FrontoParietal	0.006	0.045	0.14	0.889	1
**SensoriMotor_FrontoParietal**	**0.168**	**0.046**	**3.653**	**0.0004**	**0.014**
**Visual_FrontoParietal**	**0.137**	**0.037**	**3.688**	**0.0004**	**0.013**
Language_Language	-0.039	0.051	-0.763	0.447	1
Salience_Language	-0.01	0.042	-0.238	0.812	1
SensoriMotor_Language	0.069	0.052	1.322	0.189	1
Visual_Language	0.035	0.043	0.797	0.427	1
Salience_Salience	-0.05	0.045	-1.101	0.273	1
SensoriMotor_Salience	0.054	0.043	1.251	0.213	1
Visual_Salience	0.028	0.04	0.704	0.483	1
SensoriMotor_SensoriMotor	-0.097	0.072	-1.352	0.179	1
Visual_SensoriMotor	-0.066	0.061	-1.095	0.276	1
Visual_Visual	-0.071	0.062	-1.141	0.256	1

For Review Only
