# Supplementary material for: Brain network changes and cognitive function after cardiac arrest
Source: Brain Commun. 2024 May 23;6(4):fcae174. doi: 10.1093/braincomms/fcae174 (PMC11264146; doi:10.1093/braincomms/fcae174)
Supplement: fcae174_Supplementary_Data [file fcae174_supplementary_data.zip › Supplementary_material.pdf]

Supplementary Table 1. Neuropsychological test battery at three-month follow-up.

This table details the neuropsychological test battery used to evaluate cognitive functions in OHCA survivors at three-month follow-up. The battery assesses key cognitive domains including episodic memory, executive function, verbal fluency, and visuospatial construction. Specific tests and measures used for each cognitive domain are listed, providing a detailed overview of the assessment methodology.

Cognitive domain	Test and primary cognitive outcome	Description
**Episodic memory**	**Verbal Affective Memory Task-26 (VMT-26):**[1] Condition A7	This test evaluates the recall of emotionally charged words. Condition A7 challenges participants to remember affective words 30 minutes after learning, following an intervening task with neutral words.
**Executive function**	**Delis-Kaplan Executive System (D-KEFS):** [2]	A set of neuropsychological tests designed to assess executive functions.
	**D-KEFS Color-Word Interference Test**: Condition 4 (switching)	The test involves reading words printed in incongruent colors and requires switching between naming the word and the color. Response time and accuracy are measured.
	**D-KEFS Design Fluency Test**: Condition 3 (Switching)	Participants create unique designs by alternating between filled and empty dots. Completion time is measured.

	**D-KEFS Trail Making Test:** Condition 4 (switching)	Participants alternate between connecting numbered and lettered circles in sequence (e.g., 1 to A, A to 2). Completion time is measured.
	**D-KEFS Verbal Fluency Test**: Condition 3 (switching)	The test involves rapidly generating words based on alternating categories. Focuses on the ability to switch between categories and fluency of response.
	**Wechsler Adult Intelligence Scale (WAIS-IV):**[3] Letter-number sequencing	The test involves reordering mixed numbers and letters into ascending numerical and alphabetical order. Outcome is measured by accuracy and the ability to recall increasingly complex sequences.
**Verbal fluency**	**D-KEFS Verbal Fluency Test**: Condition 1 and 2	The test involves generating words beginning with a specific letter (Condition 1) and within a certain category (Condition 2). It focuses on lexical retrieval and category fluency.
**Visuospatial construction**	**Rey's Complex Figure Test and Recognition Trial:** [4] Conditions 3 (delayed recall) + 4 (recognition)	Condition 3 (Delayed Recall) involves reproducing a complex figure from memory after a delay. Condition 4 (Recognition) requires identifying the original figure from a set of figures.

Supplementary Table 2: Resting-state connectivity differences between OHCA survivors and healthy controls.

Comparison of within- and between-network connectivity between OHCA survivors and healthy controls, accounting for demographic variables (age, sex, and education) using Analysis of Covariance (ANCOVA). After adjusting for multiple comparisons using a post-hoc Bonferroni correction, significant increases persisted between the frontoparietal and visual networks, as well as the frontoparietal and sensorimotor networks. Bolded rows depict significant ($p<0.05$) differences.

Network1_Network2	Estimate	Standard Error	Statistic	P-value	Adjusted P-Value
Cerebellar_Cerebellar	-0.1	0.082	-1.23	0.221	1
DefaultMode_Cerebellar	-0.014	0.039	-0.367	0.715	1
DorsalAttention_Cerebellar	-0.009	0.041	-0.232	0.817	1
FrontoParietal_Cerebellar	-0.082	0.046	-1.778	0.078	1
Language_Cerebellar	-0.043	0.039	-1.098	0.274	1
Salience_Cerebellar	0	0.04	-0.012	0.99	1
SensoriMotor_Cerebellar	0.066	0.046	1.417	0.159	1
Visual_Cerebellar	0.048	0.048	0.984	0.327	1
**DefaultMode_DefaultMode**	**0.071**	**0.04**	**1.772**	**0.079**	**1**
DorsalAttention_DefaultMode	0.002	0.045	0.038	0.969	1
FrontoParietal_DefaultMode	-0.024	0.037	-0.645	0.52	1
Language_DefaultMode	0.037	0.048	0.76	0.449	1
Salience_DefaultMode	-0.025	0.049	-0.514	0.608	1
SensoriMotor_DefaultMode	0.066	0.055	1.214	0.227	1
Visual_DefaultMode	0.067	0.049	1.362	0.176	1
DorsalAttention_DorsalAttention	-0.022	0.056	-0.391	0.697	1
FrontoParietal_DorsalAttention	0.054	0.039	1.382	0.17	1
Language_DorsalAttention	0.036	0.05	0.719	0.474	1
Salience_DorsalAttention	0.007	0.037	0.185	0.853	1
SensoriMotor_DorsalAttention	0.008	0.055	0.14	0.889	1
Visual_DorsalAttention	-0.017	0.048	-0.35	0.727	1
FrontoParietal_FrontoParietal	-0.098	0.05	-1.944	0.054	1
Language_FrontoParietal	-0.022	0.041	-0.546	0.586	1

Salience_FrontoParietal	0.006	0.045	0.14	0.889	1
**SensoriMotor_FrontoParietal**	**0.168**	**0.046**	**3.653**	**0.0004**	**0.014**
**Visual_FrontoParietal**	**0.137**	**0.037**	**3.688**	**0.0004**	**0.013**
Language_Language	-0.039	0.051	-0.763	0.447	1
Salience_Language	-0.01	0.042	-0.238	0.812	1
SensoriMotor_Language	0.069	0.052	1.322	0.189	1
Visual_Language	0.035	0.043	0.797	0.427	1
Salience_Salience	-0.05	0.045	-1.101	0.273	1
SensoriMotor_Salience	0.054	0.043	1.251	0.213	1
Visual_Salience	0.028	0.04	0.704	0.483	1
SensoriMotor_SensoriMotor	-0.097	0.072	-1.352	0.179	1
Visual_SensoriMotor	-0.066	0.061	-1.095	0.276	1
Visual_Visual	-0.071	0.062	-1.141	0.256	1

Supplementary Table 3: Resting-state connectivity differences between OHCA survivors and healthy controls. Comparison of within- and between-network connectivity between OHCA survivors and healthy controls, correcting for demographic differences (age, sex, education) through propensity score weighting. After adjusting for multiple comparisons using a post-hoc Bonferroni correction significant increases persisted within the frontoparietal, dorsal attention, cerebellar and salience networks, and in between-network connectivity pertaining to the frontoparietal and cerebellar networks. Bolded rows depict significant ($p<0.05$) differences.

Network1_Network2	Estimate	Standard Error	Statistic	P-value	Adjusted p-value
**Cerebellar_Cerebellar**	**-0.2**	**0.046**	**-4.365**	**<0.0001**	**0.001**
DefaultMode_Cerebellar	-0.02	0.02	-1.39	0.167	1
DorsalAttention_Cerebellar	-0.005	0.024	-0.201	0.841	1
**FrontoParietal_Cerebellar**	**-0.151**	**0.029**	**-5.158**	**<0.0001**	**<0.0001**
Language_Cerebellar	-0.022	0.02	-1.079	0.283	1
Salience_Cerebellar	0.02	0.024	0.809	0.42	1
**SensoriMotor_Cerebellar**	**0.089**	**0.026**	**3.429**	**0.0008**	**0.031**
Visual_Cerebellar	0.017	0.026	0.658	0.512	1
DefaultMode_DefaultMode	0.011	0.022	0.507	0.613	1
DorsalAttention_DefaultMode	0.045	0.036	1.23	0.221	1
FrontoParietal_DefaultMode	0.009	0.023	0.381	0.704	1
Language_DefaultMode	0.036	0.029	1.216	0.226	1
Salience_DefaultMode	0.042	0.034	1.242	0.217	1
SensoriMotor_DefaultMode	0.063	0.034	1.863	0.065	1
Visual_DefaultMode	0.043	0.033	1.289	0.2	1
**DorsalAttention_DorsalAttention**	**-0.095**	**0.0303**	**-3.133**	**0.002**	**0.081**
FrontoParietal_DorsalAttention	0.034	0.0256	1.327	0.187	1
Language_DorsalAttention	0.003	0.0313	0.104	0.918	1
Salience_DorsalAttention	-0.038	0.0209	-1.834	0.069	1
SensoriMotor_DorsalAttention	-0.011	0.034	-0.324	0.746	1
Visual_DorsalAttention	0.001	0.037	0.034	0.973	1
**FrontoParietal_FrontoParietal**	**-0.257**	**0.039**	**-6.615**	**<0.0001**	**<0.0001**
Language_FrontoParietal	-0.014	0.024	-0.572	0.568	1
Salience_FrontoParietal	-0.031	0.026	-1.196	0.234	1
**SensoriMotor_FrontoParietal**	**0.191**	**0.032**	**5.89**	**<0.0001**	**<0.0001**
**Visual_FrontoParietal**	**0.193**	**0.027**	**7.066**	**0.0001**	**<0.0001**
Language_Language	-0.022	0.027	-0.811	0.420	1
Salience_Language	-0.041	0.023	-1.802	0.074	1

SensoriMotor_Language	0.034	0.034	1.003	0.318	1
Visual_Language	0.012	0.032	0.394	0.694	1
**Salience_Salience**	**-0.113**	**0.027**	**-4.106**	**<0.0001**	**0.003**
SensoriMotor_Salience	0.033	0.026	1.260	0.21	1
Visual_Salience	0.036	0.025	1.396	0.165	1
SensoriMotor_SensoriMotor	-0.063	0.04	-1.593	0.114	1
Visual_SensoriMotor	-0.079	0.042	-1.871	0.064	1
Visual_Visual	-0.095	0.042	-2.274	0.025	0.918

Supplementary Table 4: Variable selection across a range of lambda values in lasso regression

This table depicts the variable selection outcomes from lasso regression at five different lambda values. Each column shows the variables retained for each lambda, demonstrating consistent selection of key predictors – education, MOCA score at discharge, cardiac ejection fraction, frontoparietal – visuospatial connectivity, and sensorimotor-language connectivity. The persistence of these variables from 0.09, including the optimal lambda of 0.097, through 0.11 indicates their robustness. Lower lambdas (0.07, 0.08) result in higher retention of variables and potential overfitting risk.

**Lambda**	**0.07**	**0.08**	**0.09**	**0.1**	**0.11**
**Variable selection**	Education<br>MoCA<br>EF<br>Targeted temperature management<br>FP-VS<br>SM-Language<br>DM-Cerebellar<br>Language-Cerebellar	Education<br>MoCA<br>EF<br>FP-VS<br>SM-Language<br>DM-Cerebellar	Education<br>MoCA<br>EF<br>FP-VS<br>SM-Language	Education<br>MoCA<br>EF<br>FP-VS<br>SM-Language	Education<br>MoCA<br>EF<br>FP-VS<br>SM-Language

Abbreviations: DM- Default mode network, EF- Ejection Fraction, FP- Frontoparietal, SM-Sensorimotor, VS- Visuospatial

Supplementary Figure 1. CONN network regions of interest.

Thirty-two regions of interest were organized into eight key resting-state networks: default mode, dorsal attention, frontoparietal, language, salience, sensorimotor, visual, and cerebellar networks. This figure was adapted from an image generated and distributed by CONN, https://web.conn-toolbox.org/home.

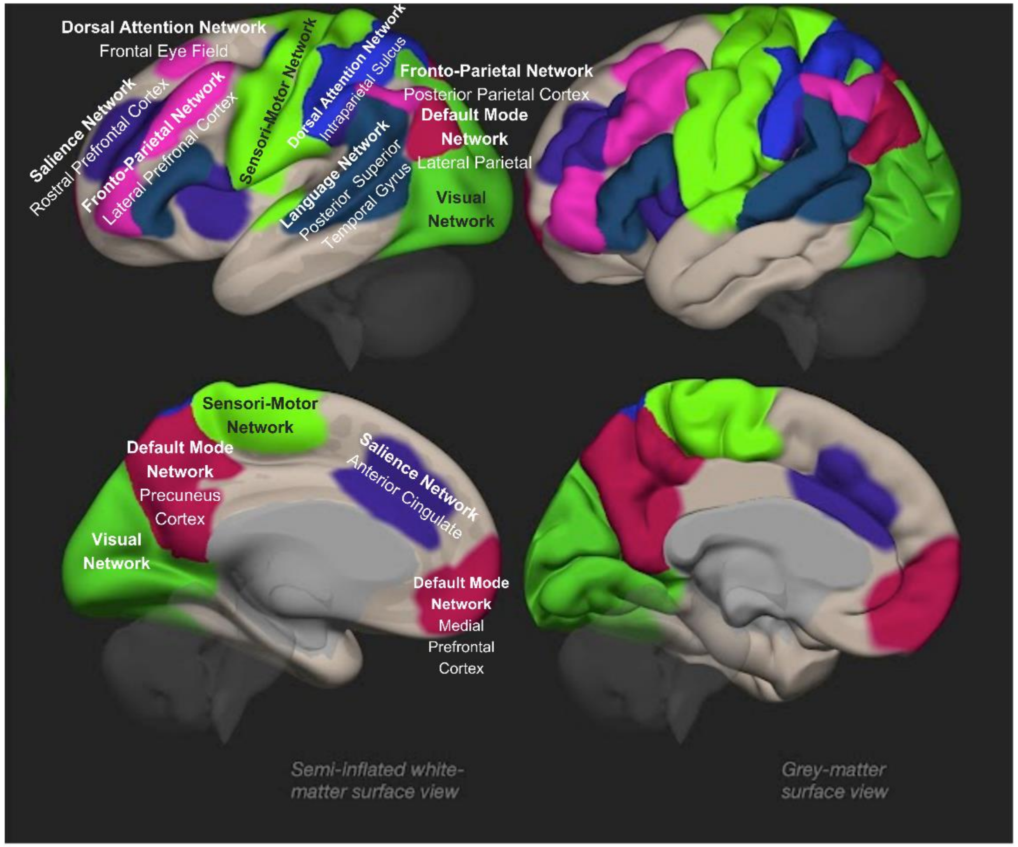

References

1. Jensen, C. G. *et al.* Development and psychometric validation of the verbal affective memory test. *Memory* **24**, 1208–1223 (2016).
2. Fine, E. M. & Delis, D. C. Delis–Kaplan Executive Functioning System. *Encyclopedia of Clinical Neuropsychology* 796–801 (2011) doi:10.1007/978-0-387-79948-3_1539.
3. Wechsler, D. (1997) Wechsler Adult Intelligence Scale. 3rd Edition, The Psychological Corporation, San Antonio. - References - Scientific Research Publishing.
4. Stern, R. A. *et al.* The Boston Qualitative Scoring System for the Rey-Osterrieth Complex Figure: Description and interrater reliability. *Clinical Neuropsychologist* **8**, 309–322 (1994).
