# Supplementary material for: Brain network changes and cognitive function after cardiac arrest
Source: Brain Commun. 2024 May 23;6(4):fcae174. doi: 10.1093/braincomms/fcae174 (PMC11264146; doi:10.1093/braincomms/fcae174)
Supplement: fcae174_Supplementary_Data [file Original Submission - Author's Response.pdf › Original Submission - Author's Response.pdf]

Below, please find item-by-item responses to the Reviewers' comments (**bolded**), which are included, and where relevant, grouped together. All page and paragraph numbers refer to locations in the submitted manuscript. Changes in the revised manuscript are highlighted in yellow when added and ~~strikethrough~~ when deleted, and a clean copy is also included.

<u>Responses to Reviewer #1</u>

1. **This is a well-written paper in an area of increasing importance as we strive to improve outcomes from OHCA.**

Thank you.

2. **Page 4: Line 41, did you select these 124 controls at random? Would it have been possible to select controls based on matched age and sex? Are there other datasets of fMRI that one could have looked at?**

We selected the 124 controls based on the availability of participants who underwent identical MRI scans at our institution. Matching on age and sex would have significantly reduced our sample size, undermining the generalizability and statistical power of the results. To maintain a larger sample size while controlling for these potentially confounding factors, we adjusted for age, sex, and education in our analyses.

To improve the comparability between the groups, we have added a more advanced propensity weighting approach under the statistical analyses (please see response number 6 to reviewer 2). We also have discussed the potential neurobiological effects of age, sex, and education on functional connectivity in lines 10-59 on page 13 and implications of our methodological approach under the Limitations.

3. **Page 5, Line 31. It appears that you used a cutoff score of more than 1.5 standard deviations. You note this as conservative. Did you consider looking at other cutoffs? Is 1.5 something that others have used?**

We recognize that the optimal methods for identifying cognitive impairments are part of a broader dialogue, with other alternatives such as 1 standard deviation for borderline cognitive impairment, or 2 standard deviations for major impairment (American Psychiatric Association. Diagnostic and statistical manual of mental disorders. 2013). We carefully selected the 1.5 standard deviation cut-off to balance sensitivity and specificity, minimizing false positive cases while capturing clinically relevant cognitive impairment. This approach is consistent with our previous work (Zarifkar et al. 2020) and supported by the heavily discussed existing literature included in the manuscript (refs 23-27).

4. **Page 7, line 42. I believe you mean AED defibrillations. Table 2 - minor typo's past cardiovascular interventions, - number of AED defibrillations Are you sure that these were all AED defibrillations and not manual defibrillations?**

Thank you for the attention to detail. We have amended "fibrillations" to "defibrillations", and corrected spacing and typographical errors in Table 2. We confirm that all instances were AED defibrillations and not manual defibrillations.

5. **The lack of asystole patients in the cohort is also worth noting briefly in the results . How was the time to ROSC defined? From EMS arrival or the time from the 911 call? Page 8, line 45, was this the initial recorded rhythm? The population you describe is young and had primarily shockable rhythms and bystander CPR suggesting that no flow times were very low. Your findings are therefore very important since this population would be expected to do very well and yet 50% had cognitive issues at 3 months. Makes one wonder how other patients who survive OHCA do cognitively.**

The described characteristics align with our cohort selection criteria focused on OHCA survivors who not only survived, but also were suitable for home discharge; a group in which asystole, longer no-flow periods and an older patient population is expected to be underrepresented due to lower survival and recovery rates. Regarding the time to ROSC, this was measured from the moment the emergency call was received.

We have updated the results section to clarify these points.

Revised, Results: "At the time of OHCA, 36 (97%) exhibited a shockable rhythm ~~and achieved ROSC within a mean of 17±15 minutes~~, with initial rhythms of ventricular fibrillation in 33 (81%), pulseless electrical activity in 4 (11%), and unknown in 3(8%). ROSC was achieved within a mean of 17±15 minutes from the emergency call."

6. **Your rate of ICD placement is also very high and deserves discussion in the limitations. Since many OHCAs are related to obstructive CAD and in these patients correcting the issue is often cited as the reason not to place an ICD. Suggest discussing this in the limitations as well since it is a form of selection bias.**

We acknowledge the reviewer's insightful observation. Indeed, ICD placement is less common in obstructive CAD cases if corrective interventions address the underlying cause, except when accompanied by low ejection fraction. Those not requiring an ICD were partly underrepresented due to quick transfers to other facilities. We have added a discussion in the limitations section of our manuscript to address the potential selection bias.

Revised, under "Limitations": "… for example, the rate of ICD placement was high (96%), potentially introducing a selection bias, where those without an indication for ICD indication after revascularization of obstructive coronary artery disease were underrepresented."

7. **Did you look at other prehospital variables that have been linked to poorer CNS functional outcomes like Epinephrine dose? Did you consider looking at no-flow and low-flow periods to see how these affected the findings? How about the public vs. private location of OHCA? Outside of education, do you have socioeconomic data that could have been looked as outcome predictors?**

In our analyses, we prioritized variables with a direct, hypothesized impact on post-OHCA cognitive outcomes, a decision driven by our study's sample size constraints. This focused approach was necessary to avoid overfitting our models. While prehospital variables like epinephrine dose, no-flow and low-flow periods, and the location of OHCA, along with socioeconomic factors beyond education, are indeed relevant, their inclusion was beyond our study's scope. We acknowledge these as potential areas for future research, especially in studies with larger cohorts that can accommodate a broader range of variables.

8. **When was the cardiac ejection fraction determined? The heart is often stunned post-cardiac arrest and the EF improves over time so when it is assessed could influence the reported value.**

To account for myocardial stunning post-arrest, cardiac ejection fraction was assessed approximately one-week post-arrest in all survivors. This timing reflects a balance between the immediate effects of cardiac arrest and practical considerations of varied hospital stays. Despite these constraints, the moderate decrease in EF observed (with only 4 performing under 40%) suggests that myocardial stunning did not significantly impact our findings. We have clarified details on the ejection fraction assessment in the manuscript.

Revised, Methods: "…cardiac ejection fraction assessed one-week post-arrest…."

9. **Figure 3, since this is a novel area of research, having a heat map of the resting connectivity in the controls, then the patients, and then the combined visual would be helpful.**

In line with the reviewer's valuable suggestion, we have included an additional heatmap that illustrates the resting-state connectivity exclusively within the control group (B), in addition to the existing heatmap exclusively within the OHCA group (A), and comparative heatmap between the OHCA group and healthy controls (C).

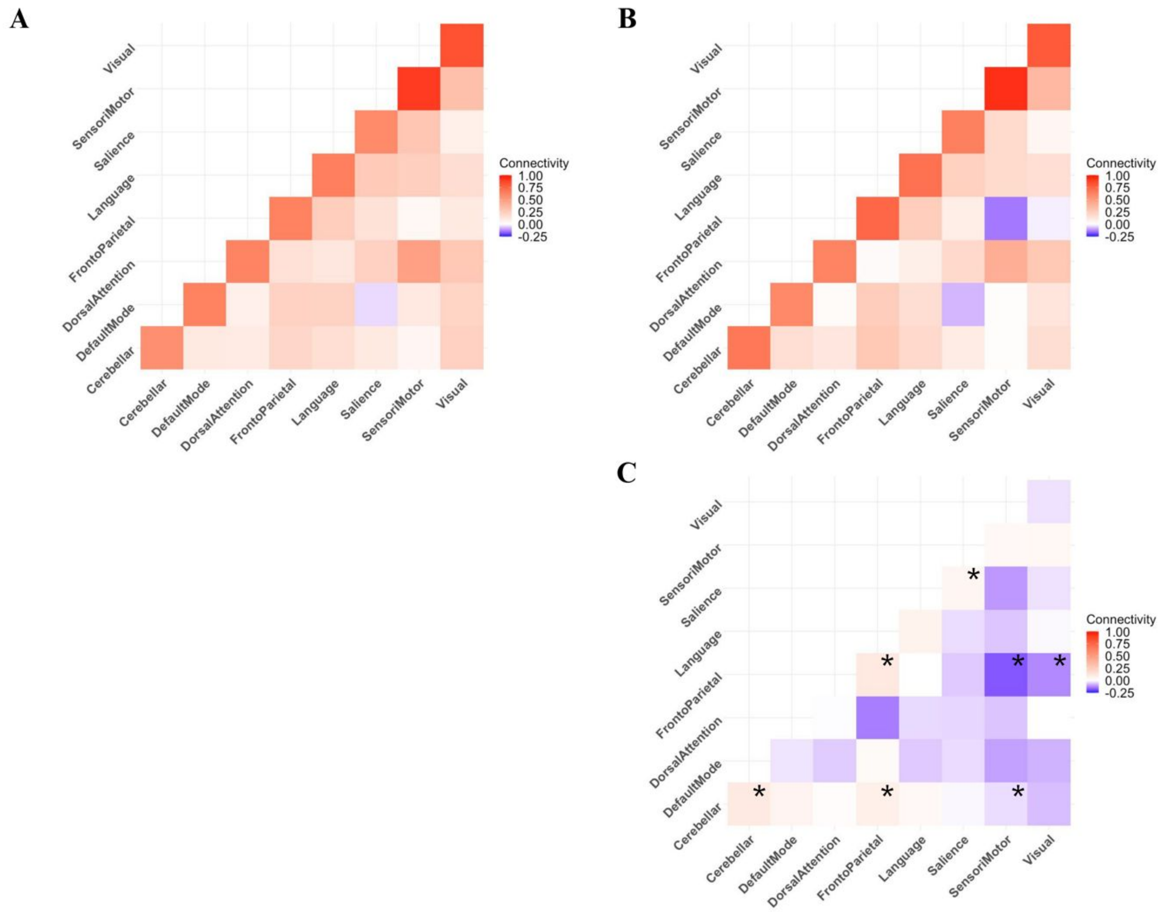

10. **Do you have any data on the post-discharge care these patients received? Was it similar? Was there any formal PT/OT for these patients that could have influenced the trajectories?**

All patients underwent a systematic interview as part of standard clinical practice two weeks post-discharge to assess needs across multiple domains (pain, physical abilities, activities of daily living, communication, sleep, mental health and cognitive functions). Based on the subjective interview, we provided personalized care plans, ensuring equal access to different rehabilitative therapy. We have detailed this approach under the Methods section.

Revised, Methods: "All patients were provided equal access to physical, psychological, and cognitive rehabilitation therapies post-discharge".

<u>Responses to Reviewer #2</u>

Comments to the Author

1. **In the research presented in the manuscript tackles the authors tackle an important question. Is it possible to use resting-state fMRI to improve the prediction of cognitive outcome in individuals recovering form out-of-hospital cardiac arrest (OHCA). This is an important question to address for a least two**

reasons. The population of OHCA survivors has been increasing, with patients often presenting with a significant burden of cognitive impairment (according to several studies cited in the manuscript about half of patients). The prediction of their outcome and the correct assessment of their need for intervention is vital. Secondly, most advanced neuroimaging methods are non-invasive and allow most patients to participate well during the examination, especially in the case of resting-state fMRI. This makes the results of the latter method especially promising for developing biomarkers for cognitive outcome in different disease states, including OHCA. I feel these facts make the study an important contribution.

I also feel the study is in many respects methodologically well grounded. The authors have made an effort to conduct a comprehensive assessment of the patients involved and have provided a good clinical description of the clinical cohort. They were also meticulous in their definition of the criteria for cognitive impairment and followed a strategy that is not likely to produce a high false positive rate of cognitive impairment (see also O'Connell et al., 2023). They also made sure to employ an fMRI resting-state acquisition sequence that help ensure a relatively reliable estimation of resting state networks (see Birn et al., 2013). All of these study characteristics make their study and the resulting manuscript a potentially promising contribution to the field.

Thank you.

2. **Nonetheless, I see the study as suffering from a major methodological limitation which all those advantages of the study do not allay – lack of statistical power. It is clear from the literature cited that large sample sizes are rare in the field. However, this does not detract from the fact that the analysis pipeline includes a huge number of statistical comparisons, potentially leading to false positive results if the appropriate use of statistical controls for that are not employed. Furthermore, the authors employed a control group which compared were poorly to the clinical group in terms of demography, significantly confounding the study results due to age and gender. Although demography and education was included in the regression analyses, this does not represent the best possible approach to the issue. Furthermore, the use of cross-validation to establish a Lambda value for LASSO regression is known the inflate type II error and in the case of the study lead to an oversight of potentially useful biomarkers for prediction (Isaksson et al., 2008; Kirpich et al., 2018). Finally, due to possible unspecified variations in the specifics of the fMRI data analysis pipeline the reproducibility of the authors results is limited, as is known form previous research (Poldrack et al., 2017; Li et al, preprint).Allow me to address in turn potential improvements in the manuscript that could help in dealing with these issues.**

These are addressed item-by-item below.

3. **When reporting adjustment for multiple comparisons it would be very helpful if the authors could be specific how these adjustments were calculated or what they**

took into account. Which multiple comparisons were taken into account? Was the number of employed psychological tests considered? How were multiple comparisons (correlations) controlled for in the CONN connectivity analysis (i.e. network-level FWE-corrected p-values; please see Alternatives to cluster-level inferences: connection-, ROI-, and network- level inferences at website https://web.conn-toolbox.org/fmri-methods/cluster-level-inferences#h.p_XkNU7W1TOYW5).

The Bonferroni correction method was used to adjust for multiple comparisons when assessing the differences in functional connectivity in the group level analyses between OHCA survivors and healthy controls. The Bonferroni method was selected for its conservative approach to reduce the risk of type I errors (false positives) in our results.

To provide transparency and allow for a comprehensive review of our findings, we included both the adjusted and unadjusted p-values in **Supplemental Table S1.** These adjustments were made using the individual connectivity results derived from the CONN toolbox, and the correction processes conducted during statistical analyses in R.

We have updated the Methods section to reflect this process more accurately.

Revised, Methods: "Post hoc ~~the~~ Bonferroni corrections ~~was~~ were applied to the group-level comparisons to adjust for multiple comparison adjustments when analyzing differences in functional connectivity between OHCA survivors and healthy controls."

Regarding the use of neuropsychological tests, we focused on the binary classification of OHCA survivors into cognitively favorable or unfavorable based on clinical cut-offs. The nature of binary classification did not necessitate adjustments for the number of neuropsychological tests because decision rule was applied once across the battery of tests.

4. **It would also be helpful if the authors rerun their LASSO regression with different Lamba values to provide the reader more insight into potential over- or underfitting, providing these results in the supplementary materials.**

We selected the optimal lambda value through cross validation as a commonly used approach because it is an objective and data driven approach to identify predictors that contribute meaningfully to the prediction task while excluding those that do not.
We agree that re-running the LASSO regression with different lambda values, and thus regularization strengths, can provide insight into the stability of the selected variables and thus improve the rigor of our analyses.

Revised, methods: "In a sensitivity analysis, we performed further LASSO regression analyses with lambda values 0.01 units above and below from the optimal value, to evaluate the stability of the selected variables."

Revised, results: “Sensitivity analyses with decrements in the lambda value included more variables, aligning with LASSO regression’s characteristics where lower lambda values carry lower penalization i.e., a reduction in the magnitude by which the regression coefficients are shrunk towards zero, resulting in higher retention of variables. Conversely, increments from the optimal lambda value consistently selected the same predictors, indicating that these variables significantly contribute to the model’s predictive ability (**Supplemental Table S4**).”

Revised, supplemental table S3: “**Supplemental Table S2: Variable selection across a range of lambda values in lasso regression**

This table depicts the variable selection outcomes from lasso regression at five different lambda values. Each column shows the variables retained for each lambda, demonstrating consistent selection of key predictors – education, MOCA score at discharge, cardiac ejection fraction, frontoparietal – visuospatial connectivity, and sensorimotor-language connectivity. The persistence of these variables from 0.09, including the optimal lambda of 0.097, through 0.11 indicates their robustness. Lower lambdas (0.07, 0.08) result in higher retention of variables and potential overfitting risk.”

**Lambda**	**0.07**	**0.08**	**0.09**	**0.1**	**0.11**
**Variable selection**	Education<br>MoCA<br>EF<br>Targeted temperature management<br>FP-VS<br>SM-Language<br>DM-Cerebellar<br>Language-Cerebellar	Education<br>MoCA<br>EF<br>FP-VS<br>SM-Language<br>DM-Cerebellar	Education<br>MoCA<br>EF<br>FP-VS<br>SM-Language	Education<br>MoCA<br>EF<br>FP-VS<br>SM-Language	Education<br>MoCA<br>EF<br>FP-VS<br>SM-Language

Abbreviations: DM- Default mode network, EF- Ejection Fraction, FP- Frontoparietal, SM- Sensorimotor, VS- Visuospatial

5. **Finally, it would also be to the advantage or readers to provide all proportions, percentages and standardized Beta regression coefficients calculated from the study data with appropriate confidence intervals. I feel this would help the readers appreciate the (limited) accuracy of findings and to allow them to put them more easily in the context of other findings.**

In striving for clarity and conciseness, we selected specific statistical measures that best illustrate the relationships and patterns identified, which are directly relevant to our study's

hypotheses. The tests included descriptive statistics, ANCOVA, and logistic regression models, among others, to evaluate the differences and predictors of cognitive outcomes post-cardiac arrest. These chosen statistics are accompanied by detailed descriptions where they provide meaningful insight into the variability and reliability of our findings and can be found throughout the manuscript as well as in the supplementary tables.

6. **To address the issue of a less than optimal control group a more advanced method of controlling for demographic variables could be employed. I would suggest that the authors employ more advanced contemporary methods of data weighting (i.e. entropic weighting) to try and address the demographic mismatch between the control and clinical groups (see Negri, 2023). It would make sense to compare the results with and without this approach.**

We appreciate the reviewer's suggestion to employ advanced propensity score weighting techniques, such as entropic weighting, to address the demographic mismatches between our control and clinical groups. Our original approach employed ANCOVA to account for potential confounding demographic variables, such as age, sex, and education. This approach is conventional and widely recognized in our field.

Follower the reviewer's recommendation, we have included propensity score weighting in our analyses as an alternative method to balance the groups.

Revised, Methods: "In sensitivity analyses, demographic matching between groups was refined using propensity score weighting, implemented through the Weightit package in R. This informed a weighted ANCOVA for functional connectivity comparisons."

Revised, Results: "The use of propensity score weighting for demographic variables confirmed increased global connectivity between resting-state networks in OHCA survivors compared to healthy controls, particularly the frontoparietal and cerebellar networks. Significant decreases in within-network connectivity were also observed for the frontoparietal, dorsal attention, cerebellar, and salience networks (**Supplemental Table S3; Figure 3).**"

Revised, Supplemental: "**Supplemental Table S3: Resting-state connectivity differences between OHCA survivors and healthy controls.** Comparison of within- and between-network connectivity between OHCA survivors and healthy controls, correcting for demographic differences (age, sex, education) through propensity score weighting. After adjusting for multiple comparisons using a post-hoc Bonferroni correction, significant increases persisted within the frontoparietal, dorsal attention, cerebellar and salience networks, and in between-network connectivity pertaining to the frontoparietal and cerebellar networks. Bolded rows depict statistically significant ($p<0.05$) differences."

7. **It would also be important provide more information on missing values and potential bias due to excluding patients. Were the 30 included in the study and 15 patients who did not participate different in any measured demographic or clinical variables.**

We agree with the reviewer's suggestion to disclose details about patients lost to follow-up. Of the 37 OHCA survivors initially included, 30 remained at follow-up, resulting in 7 dropouts. One patient was unable to participate due to a cerebral infarction during the follow-up period, while the remaining six declined to participate, two of who reported cognitive impairments. This information is illustrated in Figure 1.

Revised, Methods: "Differences in demographic and clinical characteristics between OHCA survivors and healthy controls, and between OHCA survivors at follow-up versus those lost to follow-up, were assessed using t-tests or Welch's t-tests (t), Mann-Whitney U-tests (W), or Chi-squared tests (χ^2) as appropriate."

Revised, Results: "No significant differences were observed between patients lost to follow-up and those attending follow-up in terms of age (t=-0.31, p=0.77), sex (χ^2=0.17, p=0.68), education (W=121, p=0.14), baseline MoCA scores (t=-0.10, p=0.92), or clinical characteristics (p>0.05)."

8. **The authors should also inform the reader about how many patients were excluded due to higher depression scores on Hospital Anxiety and Depression Scale or had a »psychiatric condition« (Was that assessed looking at hospital charts?). This data could easily be presented by just slightly modify Figure 1 to have separate N's for excluded patients due neurological, psychiatric and cognitive impairments.**

In the flowchart presented in Figure 1, the category "neurological, psychiatric, and cognitive impairments" denotes pre-existing conditions. Although high depression scores on the HADS scale were established as an exclusion criterion, no patients exhibited such elevated scores; consequently, none were excluded for this reason.

Revised, Figure 1 text:
~~"Neurological, psychiatric, and cognitive impairments (n=64)~~
Previous cerebrovascular or traumatic brain injury (n=39)
Dementia or untreated psychiatric disorder (n=25)"

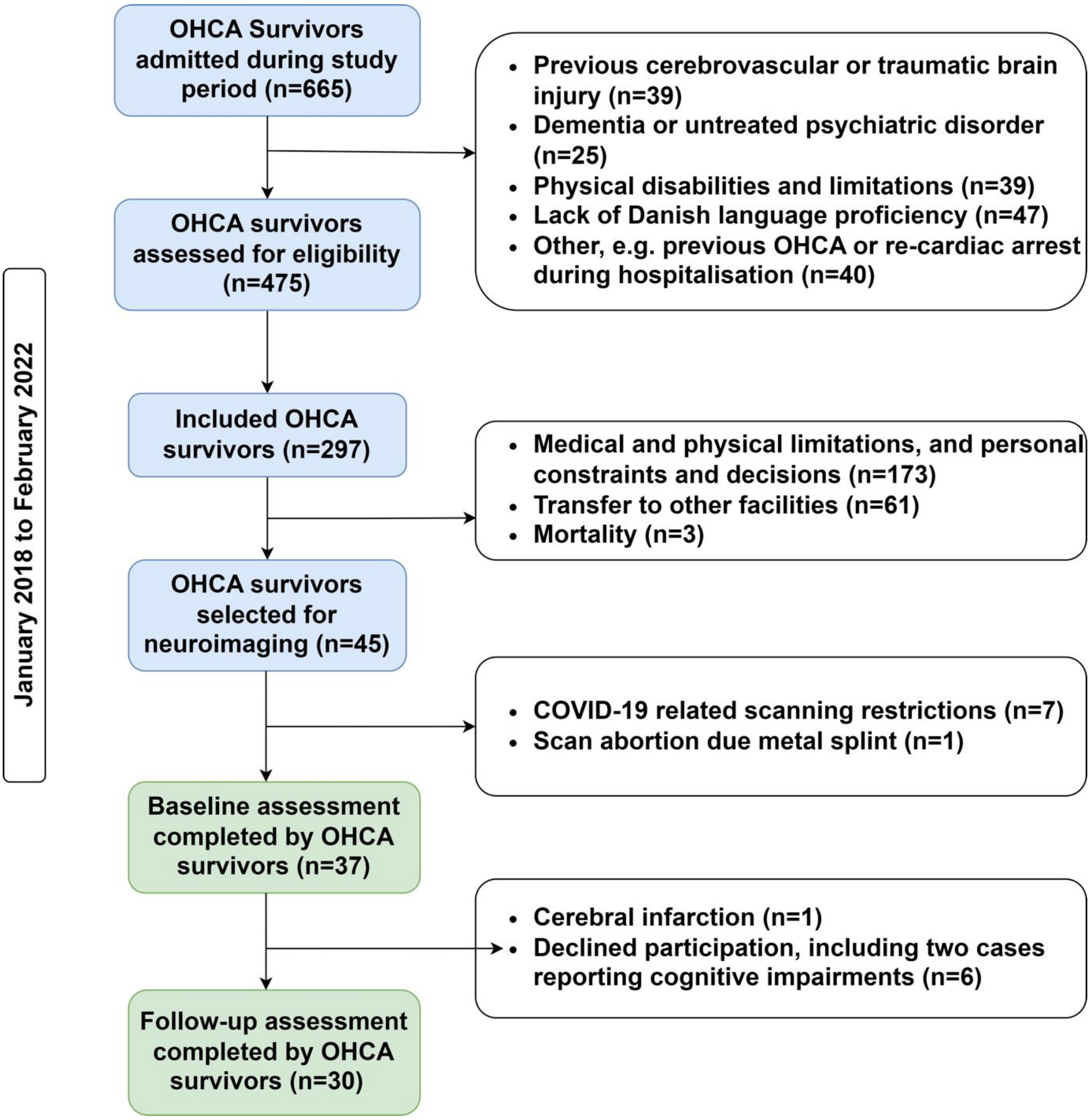

9. **In terms of study reproducibility, I would make sense to published the data analysis pipeline and all the code on a github server or similar service. This would allow readers to look up specific settings such at the type of spatial normalization that was used. I feel this should not be an issue in light of the authors willingness to provide anonymized data and analysis pipeline upon request.**

We agree with the reviewer that it is good practice to publish our analysis pipeline where it aligns with safeguarding patient data and related intellectual property. We have therefore published relevant parts of the data analysis pipeline on github. These can be found following the link: https://github.com/fishpm/revival/

10. **Finally, I also feel the manuscript would also benefit from presenting the results in terms of potential improvements in prediction accuracy if resting-state fMRI**

connectivity biomarkers are used. I would seem important to think about the obtained results not in terms of standardized beta values but other metrics such as prediction accuracy (i.e. AUC, with confidence intervals). How much more accurate can the predictions become when using fMRI biomarkers compared to clinical markers alone? 1%, 5%, 20%? Those are vital questions when looking at the economic and/or clinical implications daily practice.

The objective of this study was to examine the associations among clinical, demographic, and neuroimaging variables with cognitive outcomes in OHCA survivors. The participant number, while reasonable for our field, is too modest for robust model development and validation intended for wider populations. Our focus was to understand cognitive outcome correlates post-OHCA, offering insights that set the stage for subsequent, larger-scale research. Thus, discussing predictive accuracy metrics is beyond the scope of this study.

11. **There are also some minor issues that need to be addressed:**
    Were all the variables normalized to same scale before entering them into the LASSO regression? If not, please do so and re-check the results.

We confirm that all variables indeed were standardized before entering into the lasso regression model to ensure that all variables contribute equally to the penalty term regardless of their original scale.

12. **Please explain what the description »mean of 17±15« minutes (does 15 refer to SD, IQR or another statistical parameter) (page 8, line 47)?**

These values represented mean ± standard deviations. Relevant amendments have been made to the manuscript for clarity: standard deviation details have been removed from the main text and are now only included in Table ~~2~~1, with clear presentation of these details.

13. **When giving statistical parameters I was for which statistical parameter W stands for? Is this the Wilcoxon signed-rank test?**

W indeed stands for Wilcoxon signed-rank test (or Mann-Whitney U-test).
Statistical abbreviations have been clarified in the methods section.

Revised, Methods: ”Differences in demographic and clinical characteristics between OHCA survivors and healthy controls, and between OHCA survivors at follow-up versus those lost to follow-up, were assessed using t-tests or Welch’s t-tests (t), Mann-Whitney U-tests (W), or Chi-squared tests (χ^2) as appropriate.”

14. **Stating statistically insignificant results in the discussion seems improper »Analyses revealed distinctive brain connectivity patterns between OHCA survivors at discharge and healthy controls. OHCA survivors had fMRI results compatible with increased global network connectivity, though this difference diminished when adjusted for demographic factors« (page 12, 36-42). I feel this statement,**

especially due to the poor comparability between the control and clinical group, doesn't help in making things clearer. Either leave it out or explain it better and connect it to the discussion text on the impact of demographic factors.

Thank you for the insight. We have removed this part of the discussion to avoid confusion and entirely revised this section of the discussion.

15. **The statment »…predominantly male group of OHCA survivors who demonstrated increased network connectivity and heterogeneity...« is unclear (page 13, 50-51). What do the authors mean by "network heterogeneity". To which results (table, supplemental table or image) are the authors referring.**

We agree with the reviewer that the use of the word heterogeneity in this context may carry several interpretations. In this context, we were referring to heterogeneity in the strength and direction of connectivity between networks across the two groups, as determined through a visual inspection of Figure 3 and accompany values in supplemental Table S2.

Revised, discussion: "Given these findings, ~~the younger, well-educated control group with a balanced sex distribution would be expected to have higher network connectivity . However, contrary to these expectations, it was~~ the older, less educated, and predominantly male group of OHCA survivors would be expected to have lower global network connectivity, with a pattern of functional integration and segregation centered on the default mode network. ~~who demonstrated increased network connectivity and heterogeneity.~~ Contrary to these expectations, OHCA survivors had a distinct pattern of functional reorganization centered on the frontoparietal network, with unchanged global connectivity."

16. **Table 1. Why is verbal fluency seperate from executive function, since many would consider verbal fluency based on a phonetic cue to be a executive functioning task?**

We recognize the overlap among neuropsychological domains for many neuropsychological tests. Therefore, tests that involve multiple cognitive domains were placed under the domain most relevant to their primary cognitive demand in our analysis. In our study, we differentiated the D-KEFS verbal fluency test conditions based on their primary cognitive requirements. Conditions 1 and 2 were categorized together to emphasize the core aspect of verbal fluency, while Condition 3 was classified under executive functions due to its heightened demand for executive control processes.

17. **How was the p-adjusted calcualted in Supplemental Table 1?**

This was based on the Bonferroni correction. The figure legend has been updated for clarity.

Revised, Supplemental Table ~~1~~2 legend: "After adjusting for multiple comparisons using a post-hoc Bonferroni correction, …."

18. **An image of the »networks« CONN Atlas such as the one below woudl be helpful.**

Revised, Figure: “**Supplemental Figure 1. CONN network regions of interest.** **Supplemental Figure 1. CONN network regions of interest.**

Thirty-two regions of interest were organized into eight key resting-state networks: default mode, dorsal attention, frontoparietal, language, salience, sensorimotor, visual, and cerebellar networks. This image was generated and distributed by CONN, https://web.conn-toolbox.org/home.”

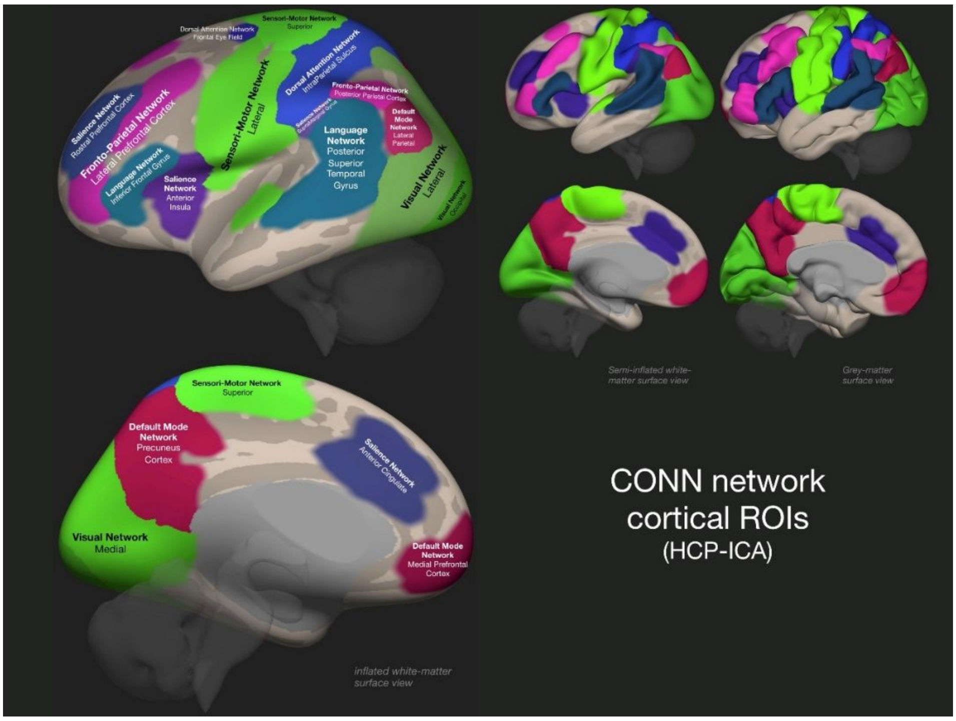

Literature:

O’Connell, M., Kadlec, H., Griffith, L. et al. Cognitive impairment indicator for the neuropsychological test batteries in the Canadian Longitudinal Study on Aging: definition and evidence for validity. Alz Res Therapy 15, 167 (2023). https://doi.org/10.1186/s13195-023-01317-3

Birn RM, Molloy EK, Patriat R, Parker T, Meier TB, Kirk GR, Nair VA, Meyerand ME, Prabhakaran V. The effect of scan length on the reliability of resting-state fMRI connectivity estimates. Neuroimage. 2013 Dec;83:550-8. doi: 10.1016/j.neuroimage.2013.05.099. Epub 2013 Jun 6. PMID: 23747458; PMCID: PMC4104183.

Isaksson A, Wallman M, Go ransson H, Gustafsson MG. Cross-validation and bootstrapping are unreli- able in small sample classification. Pattern Recognition Letters. 2008; 29(14):1960–1965. https://doi. org/10.1016/j.patrec.2008.06.018

Kirpich A, Ainsworth EA, Wedow JM, Newman JRB, Michailidis G, McIntyre LM (2018) Variable selection in omics data: A practical evaluation of small sample sizes. PLoS ONE 13(6): e0197910. https://doi.org/10.1371/journal. pone.0197910

Poldrack, R. A., Baker, C. I., Durnez, J., Gorgolewski, K. J., Matthews, P. M., Munafo, M. R., . . . Yarkoni, T. (2017). Scanning the horizon: towards transparent and reproducible neuroimaging research. Nature Reviews Neuroscience, 18(2), 115-126. doi: 10.1038/nrn.2016.167

Moving Beyond Processing and Analysis-Related Variation in Neuroscienc (2024). Xinhui Li, Nathalia Bianchini Esper, Lei Ai, Steve Giavasis, Hecheng Jin, Eric Feczko, Ting Xu, Jon Clucas, Alexandre Franco, Anibal Sólon Heinsfeld, Azeez Adebimpe, Joshua T. Vogelstein, Chao-Gan Yan, Oscar Esteban, Russell A. Poldrack, Cameron Craddock, Damien Fair, Theodore Satterthwaite, Gregory Kiar, Michael P. Milham, bioRxiv 2021.12.01.470790; doi: https://doi.org/10.1101/2021.12.01.470790

Negri, F. (2023). Correlation Is Not Causation, Yet… Matching and Weighting for Better Counterfactuals. In: Damonte, A., Negri, F. (eds) Causality in Policy Studies. Texts in Quantitative Political Analysis. Springer, Cham. https://doi.org/10.1007/978-3-031-12982-7_4

Comments from the Scientific Editorial team:

-Please supply a graphical abstract. A graphical abstract is a visual representation of the central finding or methodology of the paper. It will be displayed in the table of contents. Authors should strive to make the graphical abstract informative, interesting, visually appealing, and straightforward (no caption needed, no abbreviations except gene names). The technical requirements for the graphical abstracts are:
- Size: 120 x 120 mm (300-600dpi) or 3000 x 3000 pixels
- Font: Arial 12-16 pt
- File type: tiff or PDFdat
- Aspect ratio: 1:1 preferred

-Please add an Abbreviated Summary (up to 50 words) of your paper that captures the main purpose and conclusions of the work. The summary should state the main results written in the third person (‘[1st author surname] et al. report that …’) and conclusion. This summary will be used in the contents list online and should not contain any abbreviations other than those on the accepted abbreviations list and accepted gene/protein names, making it accessible to the non-specialist. Please upload it as a Word document. Allowed abbreviations: AIDS; ANOVA; AMPA; ATP; A,T,C,G; CNS; CSF; CT; DNA; ECG; EEG; EMG; GABA; HIV; MRI; NMDA; PET; PCR; RNA.

-Please include the appropriate checklist to comply with our ethical standards (see Reporting Guideline section within 'Instructions to authors').

-Data availability: Please provide the codes generated and used in this work within the supplementary material or through a repository, whose link has to be made available within the data availability statement.

-Tables: We noticed that tables 1 and 2 are likely to exceed one journal page. Large tables that are more than one page in length, particularly in landscape orientation, are difficult to read and are not usually allowed; such tables should be uploaded as Supplementary material. Supplementary tables containing large amounts of text can be summarized in a smaller table for publication in the article if appropriate. Alternatively, you could consider splitting a large table into smaller tables. Please use the attached template to format your tables, and to check whether they are likely to exceed one typeset journal page.

-Figures 2 and 3: Please ensure that subfigures are formatted according to journal guidelines- Use upper case letters, A, B, C, etc, to label panels in top left corner (no brackets or full stops).

-Figure 2: For each analysis, please report the statistical test used and state the p-values obtained from the statistical tests in the figure legend. Also, please label the axes.

-Figure 3: If applicable, please report the statistical test used in the figure legend.

All edits following comments from the Scientific Editorial team have been made to the manuscript.
