# Supplementary material for: Brain network changes and cognitive function after cardiac arrest
Source: Brain Commun. 2024 May 23;6(4):fcae174. doi: 10.1093/braincomms/fcae174 (PMC11264146; doi:10.1093/braincomms/fcae174)
Supplement: fcae174_Supplementary_Data [file Original Submission - Decision Letter.pdf › Original Submission - Decision Letter.pdf]

Original Submission - Decision Letter

29-Feb-2024

Dear Dr. Hassager,

Thank you for your recent submission BRAINCOM-2024-010 entitled "Brain network changes and cognitive function after cardiac arrest" to Brain Communications.

Your manuscript has been reviewed, and while the reviewers and editorial team appreciate many aspects of the paper, they raise some concerns that you will need to address in order for the paper to be considered for publication.

If you can address these concerns, I invite you to submit a revised manuscript and a response to each of the reviewers' comments. The reviewer(s)' comments are included at the bottom of this message.

In addition to the reviewers' comments, please also address our Scientific Editor's comments added below to comply with our requirements for presentation of scientific data at Brain Communications.

Please format your revised manuscript according to our Instructions to Authors https://academic.oup.com/braincomms/pages/General_Instructions
A summary of key points is attached to help with this. Please note that there can be substantial delays to publication if you don't resubmit in line with our requirements.

To revise your manuscript, click the link below or log into https://mc.manuscriptcentral.com/braincom and enter your Author Centre, where you will find your manuscript title listed under "Manuscripts with Decisions." Under "Actions," click on "Create a Revision." Your manuscript number will be appended to denote a revision.

*** PLEASE NOTE: This is a two-step process. After clicking on the link, you will be directed to a webpage to confirm. ***

https://mc.manuscriptcentral.com/braincom?URL_MASK=95c08cbf47f241dc924a05e44c2ee363

Please upload a revised manuscript with changes tracked with track changes or in coloured text.

When submitting your revised manuscript, you will be asked to respond to the reviewer(s) comments. Please be as specific as possible in your response to all of the comments.

The editorial team at Brain Communications aims to increase transparency in scientific publishing and to demonstrate the value that peer review brings to the scientific process. Please therefore consider whether you are willing to have reviewer(s)' comments on your paper and your response published as supplementary data with the paper if it is accepted. This is entirely optional and will only be done if both the authors and reviewers agree. While submitting your revision there will be an opportunity to review or update the answer you gave previously, if necessary.

IMPORTANT: Your original files are available to you when you upload your revised manuscript. Please delete any redundant files before completing the submission.

Because we are trying to facilitate timely publication of manuscripts submitted to Brain Communications, we would appreciate your revised manuscript as soon as possible. If it is not possible for you to resubmit within 3 months, the option to submit a revision will expire, and we would then consider your revised paper as a new submission. However, if you need a little more time, please get in touch and we can extend this deadline for you.

Once again, thank you for submitting your manuscript to Brain Communications and I look forward to receiving your revision.

Best wishes

Dr. Grega Repovs
Associate Editor, Brain Communications

Professor Tara Spires-Jones
Founding Editor, Brain Communications

Reviewer(s)' Comments to Author:

Reviewer: 1

Comments to the Author
This is a well-written paper in an area of increasing importance as we strive to improve outcomes from OHCA.

Page 4: Line 41, did you select these 124 controls at random? Would it have been possible to select controls based on matched age and sex? Are there other datasets of fMRI that one could have looked at?

Page 5, Line 31. It appears that you used a cutoff score of more than 1.5 standard deviations. You note this as conservative. Did you consider looking at other cutoffs? Is 1.5 something that others have used?

Page 7, line 42. I believe you mean AED defibrillations. Did you look at other prehospital variables that have been linked to poorer CNS functional outcomes like Epinephrine dose? How was the time to ROSC defined? From EMS arrival or the time from the 911 call? Did you consider looking at no-flow and low-flow periods to see how these affected the findings? How about the public vs. private location of OHCA? When was the cardiac ejection fraction determined? The heart is often stunned post-cardiac arrest and the EF improves over time so when it is assessed could influence the reported value.

Page 8, line 45, was this the initial recorded rhythm? The population you describe is young and had primarily shockable rhythms and bystander CPR suggesting that no flow times were very low. Your findings are therefore very important since this population would be expected to do very well and yet 50% had cognitive issues at 3 months. Makes one wonder how other patients who survive OHCA do cognitively. Your rate of ICD placement is also very high and deserves discussion in the limitations. Since many OHCAs are related to obstructive CAD and in these patients correcting the issue is often cited as the reason not to place an ICD. Suggest discussing this in the limitations as well since it is a form of selection bias.

Table 2 - minor typo's past cardiovascular interventions, - number of AED defibrillations Are you sure that these were all AED defibrillations and not manual defibrillations?The lack of asystole patients in the cohort is also worth noting briefly in the results.

Figure 3, since this is a novel area of research, having a heat map of the resting connectivity in the controls, then the patients, and then the combined visual would be helpful.

Do you have any data on the post-discharge care these patients received? Was it similar? Was there any formal PT/OT for these patients that could have influenced the trajectories? Outside of education, do you have socioeconomic data that could have been looked as outcome predictors?

Reviewer: 2 (David Gosar)

Comments to the Author

In the research presented in the manuscript tackles the authors tackle an important question. Is it possible to use resting-state fMRI to improve the prediction of cognitive outcome in individuals recovering form out-of-hospital cardiac arrest (OHCA). This is an important question to address for a least two reasons. The population of OHCA survivors has been increasing, with patients often presenting with a significant burden of cognitive impairment (according to several studies cited in the manuscript about half of patients). The prediction of their outcome and the correct assessment of their need for intervention is vital. Secondly, most advanced neuroimaging methods are non-invasive and allow most patients to participate well during the examination, especially in the case of resting-state fMRI. This makes the results of the latter method especially promising for developing biomarkers for cognitive outcome in different disease states, including OHCA. I feel these facts make the study an important contribution.

I also feel the study is in many respects methodologically well grounded. The authors have made an effort to conduct a comprehensive assessment of the patients involved and have provided a good clinical description of the clinical cohort. They were also meticulous in their definition of the criteria for cognitive impairment and followed a strategy that is not likely to produce a high false positive rate of cognitive impairment (see also O'Connell et al., 2023). They also made sure to employ an fMRI resting-state acquisition sequence that help ensure a relatively reliable estimation of resting state networks (see Birn et al., 2013). All of these study characteristics make their study and the resulting manuscript a potentially promising contribution to the field.

Nonetheless, I see the study as suffering from a major methodological limitation which all those advantages of the study do not allay – lack of statistical power. It is clear from the literature cited that large sample sizes are rare in the field. However, this does not detract from the fact that the analysis pipeline includes a huge number of statistical comparisons, potentially leading to false positive results if the appropriate use of statistical controls for that are not employed. Furthermore, the authors employed a control group which compared were poorly to the clinical group in terms of demography, significantly confounding the study results due to age and gender. Although demography and education was included in the regression analyses, this does not represent the best possible approach to the issue. Furthermore, the use of cross-validation to establish a Lambda value for LASSO regression is known the inflate type II error and in the case of the study lead to an oversight of potentially useful biomarkers for prediction (Isaksson et al., 2008; Kirpich et al., 2018). Finally, due to possible unspecified variations in the specifics of the fMRI data analysis pipeline the reproducibility of the authors results is limited, as is known form previous research (Poldrack et al., 2017; Li et al, preprint).

Allow me to address in turn potential improvements in the manuscript that could help in dealing with these issues. When reporting adjustment for multiple comparisons it would be very helpful if the authors could be specific how these adjustments were calculated or what they took into account. Which multiple comparisons were taken into account? Was the number of employed psychological tests considered? How were multiple comparisons (correlations) controlled for in the CONN connectivity analysis (i.e. network-level FWE-corrected p-values; please see Alternatives to cluster-level inferences: connection-, ROI-, and network- level inferences at website https://web.conn-toolbox.org/fmri-methods/cluster-level-inferences#h.p_XkNU7W1TOYW5). It would also be helpful if the authors rerun their LASSO regression with different Lamba values to provide the reader more insight into potential over- or underfitting, providing these results in the supplementary materials. Finally, it would also be to the advantage or readers to provide all proportions, percentages and standardized Beta regression coefficients calculated from the study data with appropriate confidence intervals. I feel this would help the readers appreciate the (limited) accuracy of findings and to allow them to put them more easily in the context of other findings.

To address the issue of a less than optimal control group a more advanced method of controlling for demographic variables could be employed. I would suggest that the authors employ more advanced contemporary methods of data weighting (i.e. entropic weighting) to try and address the demographic mismatch between the control and clinical groups (see Negri, 2023). It would make sense to compare the

results with and without this approach. It would also be important provide more information on missing values and potential bias due to excluding patients. Were the 30 included in the study and 15 patients who did not participate different in any measured demographic or clinical variables. The authors should also inform the reader about how many patients were excluded due to higher depression scores on Hospital Anxiety and Depression Scale or had a »psychiatric condition« (Was that assessed looking at hospital charts?). This data could easily be presented by just slightly modify Figure 1 to have separate N's for excluded patients due neurological, psychiatric and cognitive impairments.

In terms of study reproducibility, I would make sense to published the data analysis pipeline and all the code on a github server or similar service. This would allow readers to look up specific settings such at the type of spatial normalization that was used. I feel this should not be an issue in light of the authors willingness to provide anonymized data and analysis pipeline upon request.

Finally, I also feel the manuscript would also benefit from presenting the results in terms of potential improvements in prediction accuracy if resting-state fMRI connectivity biomarkers are used. I would seem important to think about the obtained results not in terms of standardized beta values but other metrics such as prediction accuracy (i.e. AUC, with confidence intervals). How much more accurate can the predictions become when using fMRI biomarkers compared to clinical markers alone? 1%, 5%, 20%? Those are vital questions when looking at the economic and/or clinical implications daily practice.

There are also some minor issues that need to be addressed:

- Were all the variables normalized to same scale before entering them into the LASSO regression? If not, please do so and re-check the results.
- Please explain what the description »mean of 17±15« minutes (does 15 refer to SD, IQR or another statistical parameter) (page 8, line 47)?
- When giving statistical parameters I was for which statistical parameter W stands for? Is this the Wilcoxon signed-rank test?
- Stating statistically insignificant results in the discussion seems improper »Analyses revealed distinctive brain connectivity patterns between OHCA survivors at discharge and healthy controls. OHCA survivors had fMRI results compatible with increased global network connectivity, though this difference diminished when adjusted for demographic factors« (page 12, 36-42). I feel this statement, especially due to the poor comparability between the control and clinical group, doesn’t help in making things clearer. Either leave it out or explain it better and connect it to the discussion text on the impact of demographic factors.
- The statment »…predominantly male group of OHCA survivors who demonstrated increased network connectivity and heterogeneity...« is unclear (page 13, 50-51). What do the authors mean by “network heterogeneity”. To which results (table, supplemental table or image) are the authors referring.
- Table 1. Why is verbal fluency seperate from executive function, since many would consider verbal fluency based on a phonetic cue to be a executive functioning task?
- How was the p-adjusted calcualted in Supplemental Table 1?
- An image of the »networks« CONN Atlas such as the one below woudl be helpful.

Literature:

O’Connell, M., Kadlec, H., Griffith, L. et al. Cognitive impairment indicator for the neuropsychological test batteries in the Canadian Longitudinal Study on Aging: definition and evidence for validity. Alz Res Therapy 15, 167 (2023). https://doi.org/10.1186/s13195-023-01317-3

Birn RM, Molloy EK, Patriat R, Parker T, Meier TB, Kirk GR, Nair VA, Meyerand ME, Prabhakaran V. The effect of scan length on the reliability of resting-state fMRI connectivity estimates. Neuroimage. 2013 Dec;83:550-8. doi: 10.1016/j.neuroimage.2013.05.099. Epub 2013 Jun 6. PMID: 23747458; PMCID: PMC4104183.

Isaksson A, Wallman M, Go¨ransson H, Gustafsson MG. Cross-validation and bootstrapping are unreliable in small sample classification. Pattern Recognition Letters. 2008; 29(14):1960–1965. https://doi.org/10.1016/j.patrec.2008.06.018

Kirpich A, Ainsworth EA, Wedow JM, Newman JRB, Michailidis G, McIntyre LM (2018) Variable selection in omics data: A practical evaluation of small sample sizes. PLoS ONE 13(6): e0197910. https://doi.org/10.1371/journal. pone.0197910

Poldrack, R. A., Baker, C. I., Durnez, J., Gorgolewski, K. J., Matthews, P. M., Munafo, M. R., . . . Yarkoni, T. (2017). Scanning the horizon: towards transparent and reproducible neuroimaging research. Nature Reviews Neuroscience, 18(2), 115-126. doi: 10.1038/nrn.2016.167

Moving Beyond Processing and Analysis-Related Variation in Neuroscienc (2024). Xinhui Li, Nathalia Bianchini Esper, Lei Ai, Steve Giavasis, Hecheng Jin, Eric Feczko, Ting Xu, Jon Clucas, Alexandre Franco, Anibal Sólon Heinsfeld, Azeez Adebimpe, Joshua T. Vogelstein, Chao-Gan Yan, Oscar Esteban, Russell A. Poldrack, Cameron Craddock, Damien Fair, Theodore Satterthwaite, Gregory Kiar, Michael P. Milham, bioRxiv 2021.12.01.470790; doi: https://doi.org/10.1101/2021.12.01.470790

Negri, F. (2023). Correlation Is Not Causation, Yet… Matching and Weighting for Better Counterfactuals. In: Damonte, A., Negri, F. (eds) Causality in Policy Studies. Texts in Quantitative Political Analysis. Springer, Cham. https://doi.org/10.1007/978-3-031-12982-7_4

Comments from the Scientific Editorial team:

-Please supply a graphical abstract. A graphical abstract is a visual representation of the central finding or methodology of the paper. It will be displayed in the table of contents. Authors should strive to make the graphical abstract informative, interesting, visually appealing, and straightforward (no caption needed, no abbreviations except gene names). The technical requirements for the graphical abstracts are:
- Size: 120 x 120 mm (300-600dpi) or 3000 x 3000 pixels
- Font: Arial 12-16 pt
- File type: tiff or PDFdat
- Aspect ratio: 1:1 preferred

-Please add an Abbreviated Summary (up to 50 words) of your paper that captures the main purpose and conclusions of the work. The summary should state the main results written in the third person (‘[1st author surname] et al. report that …’) and conclusion. This summary will be used in the contents list online and should not contain any abbreviations other than those on the accepted abbreviations list and accepted gene/protein names, making it accessible to the non-specialist. Please upload it as a Word document. Allowed abbreviations: AIDS; ANOVA; AMPA; ATP; A,T,C,G; CNS; CSF; CT; DNA; ECG; EEG; EMG; GABA; HIV; MRI; NMDA; PET; PCR; RNA.

-Please include the appropriate checklist to comply with our ethical standards (see Reporting Guideline section within 'Instructions to authors').

-Data availability: Please provide the codes generated and used in this work within the supplementary material or through a repository, whose link has to be made available within the data availability statement.

-Tables: We noticed that tables 1 and 2 are likely to exceed one journal page. Large tables that are more than one page in length, particularly in landscape orientation, are difficult to read and are not usually allowed; such tables should be uploaded as Supplementary material. Supplementary tables containing large amounts of text can be summarized in a smaller table for publication in the article if appropriate. Alternatively, you could consider splitting a large table into smaller tables. Please use the attached template to format your tables, and to check whether they are likely to exceed one typeset journal page.

-Figures 2 and 3: Please ensure that subfigures are formatted according to journal guidelines- Use upper case letters, A, B, C, etc, to label panels in top left corner (no brackets or full stops).

-Figure 2: For each analysis, please report the statistical test used and state the p-values obtained from the statistical tests in the figure legend. Also, please label the axes.

-Figure 3: If applicable, please report the statistical test used in the figure legend.
