# Supplementary material for: Brain network changes and cognitive function after cardiac arrest
Source: Brain Commun. 2024 May 23;6(4):fcae174. doi: 10.1093/braincomms/fcae174 (PMC11264146; doi:10.1093/braincomms/fcae174)
Supplement: fcae174_Supplementary_Data [file Revision 1 - Author's Response.pdf › Revision 1 - Author's Response.pdf]

Response to reviewer:

Thank you to the reviewers and editorial team for the provisional acceptance of our manuscript. Figure 2 and its legend have been revised according to the Scientific Editor team's request. Table 1 has been revised to fit within the margins of one A4 page.
