# Supplementary material for: Brain network changes and cognitive function after cardiac arrest
Source: Brain Commun. 2024 May 23;6(4):fcae174. doi: 10.1093/braincomms/fcae174 (PMC11264146; doi:10.1093/braincomms/fcae174)
Supplement: fcae174_Supplementary_Data [file Revision 1 - Decision Letter.pdf › Revision 1 - Decision Letter.pdf]

Revision 1 - Decision Letter

15-Apr-2024

Dear Dr. Hassager,

Thank you for your recent submission BRAINCOM-2024-010.R1
entitled "Brain network changes and cognitive function after cardiac arrest" to Brain Communications.

I am pleased to let you know that we would like to provisionally accept your manuscript entitled "Brain network changes and cognitive function after cardiac arrest" for publication in Brain Communications. There are still some minor points for you to address before final acceptance, but further peer-review will not be necessary.
Please would you respond to the comments shown below from reviewers and/or our Scientific Editor (regarding our requirements for presentation of scientific data at Brain Communications) and submit a revised version.

Please format your revised manuscript according to our Instructions to Authors https://academic.oup.com/braincomms/pages/General_Instructions
A summary of key points is attached to help with this. Please note that there can be substantial delays to publication if you don't resubmit in line with our requirements.

To revise your manuscript, click the link below or log into https://mc.manuscriptcentral.com/braincom and enter your Author Centre, where you will find your manuscript title listed under "Manuscripts with Decisions." Under "Actions," click on "Create a Revision." Your manuscript number will be appended to denote a revision.

*** PLEASE NOTE: This is a two-step process. After clicking on the link, you will be directed to a webpage to confirm. ***

https://mc.manuscriptcentral.com/braincom?URL_MASK=a8c478ca85314153acdf61f88e34d77b

Please upload a revised manuscript with the changes you have made shown either with "track changes" or in coloured text.

When submitting your revised manuscript, you will be asked to respond to the reviewer(s) and editor's comments. Please be as specific as possible in your response to all of the comments.

The editorial team at Brain Communications also aims to increase transparency in scientific publishing and to demonstrate the value that peer review brings to the scientific process. Please therefore consider whether you are willing to have reviewer comments on your paper and your response published as supplementary data with the paper if it is accepted. This is entirely optional and will only be done if both the authors and reviewers agree. When submitting your revision you will have the opportunity to update the answer you entered previously.

IMPORTANT: Your original files are available to you when you upload your revised manuscript. Please delete any redundant files before completing the submission.

Because we are trying to facilitate timely publication of manuscripts submitted to Brain Communications, we would appreciate your revised manuscript as soon as possible. If it is not possible for you to submit your revision within 3 months, the option to submit a revision will expire, and we will consider your revised paper as a new submission. However, if you need a little more time, please get in touch and we can extend this deadline for you.

Once again, thank you for submitting your manuscript to Brain Communications and I look forward to receiving your revision.

Best wishes

Dr. Manuela Marescotti
Scientific Editor, Brain Communications

Professor Tara Spires-Jones
Founding Editor, Brain Communications

Reviewer(s)' Comments to Author:

Reviewer: 2 (David Gosar)

Comments to the Author
I feel the authors have made sufficient effort to address the issues highlighted. I thank them for their efforts and congratulate them for their work.

Comments from the Scientific Editor:

Thank you for addressing the majority of the queries raised by the Scientific Editor team.

- Fig2a: report in the legend the statistical analysis result.

- Fig2: please label the y axes.

- Please, make sure that each table fits one A4 page.
